# Enhanced endosomal escape for nanoparticle-enabled co-delivery of doxorubicin and siRNA to overcome multidrug resistance

**DOI:** 10.1016/j.mtadv.2026.100845

**Published:** 2026-06-09

**Authors:** Jin Zhai, Allison Surian, Trystin Cote, Wuxia Zhang, Bao-Toan Dang, Yuan Wang, Qianyu Chen, Kazunori Hoshino, Jinhyung Lee, Yupeng Chen

**Affiliations:** Department of Biomedical Engineering, University of Connecticut, Storrs, CT, 06269, USA

**Keywords:** Nanoparticle, Multidrug resistance, Co-delivery, Anticancer therapy, Endosomal escape

## Abstract

Although co-delivery of small-molecule drugs and siRNAs is a promising platform strategy for combination therapy, current delivery systems fail to achieve efficient endosomal escape, limiting cytosolic siRNA bioavailability and therapeutic efficacy. Conventional lipid nanoparticles (LNPs) can co-encapsulate chemotherapeutics and siRNA, but their poor endosomal escape results in suboptimal siRNA bioavailability. Here, we report a novel type of sphere-like nanoparticle (SNP) platform for co-delivery of siRNA and small-molecule drugs that overcomes these limitations. In this system, doxorubicin (DOX) is intercalated within a Janus base nanotube (JBNt) structure, while siRNA is encapsulated through electrostatic interactions, enabling stable co-packaging. Notably, SNPs exhibit significantly enhanced endosomal escape compared with lipid nanoparticles, leveraging JBNt’s endosomal escape, attributable to its distinct proton-sponge-mediated buffer capacity, consequently promoting efficient, coordinated cytosolic delivery of both cargos. In a proof-of-concept study, SNP-mediated co-delivery of Multidrug Resistance 1 gene (MDR1/ABCB1)-targeting siRNA and DOX was associated with effective gene silencing and enhanced apoptosis in cancer cells, tumor spheroids, and murine ovarian tumor xenograft models. Collectively, these findings deliver proof-of-concept evidence for SNPs as a promising co-delivery platform for RNA and chemotherapeutics to overcome chemoresistance and improve anticancer efficacy.

## 1. Introduction

Co-delivery of small interfering RNA (siRNA) and small-molecule chemotherapeutics represents a promising strategy for combination cancer therapy, enabling simultaneous modulation of disease-driving pathways and direct cytotoxicity [1,2]. However, clinical translation of siRNA-based combination therapies has been hindered by the intrinsic limitations of RNA molecules, including nuclease susceptibility and inefficient intracellular delivery [3]. Among these barriers, endosomal entrapment remains particularly critical: efficient RNA interference (RNAi) requires cytosolic delivery of RNA cargos and timely escape from endosomal compartments to avoid lysosomal degradation [3,4]. Current delivery platforms, including lipid nanoparticles (LNPs) and cationic polymers, generally exhibit limited endosomal escape efficiency, with the majority of internalized siRNA remaining trapped and functionally inactive [5–7]. Consequently, there is an urgent need for delivery systems that enable efficient endosomal escape, while maintaining stable co-packaging of nucleic acid and small-molecule cargos.

**Fig. 1.**
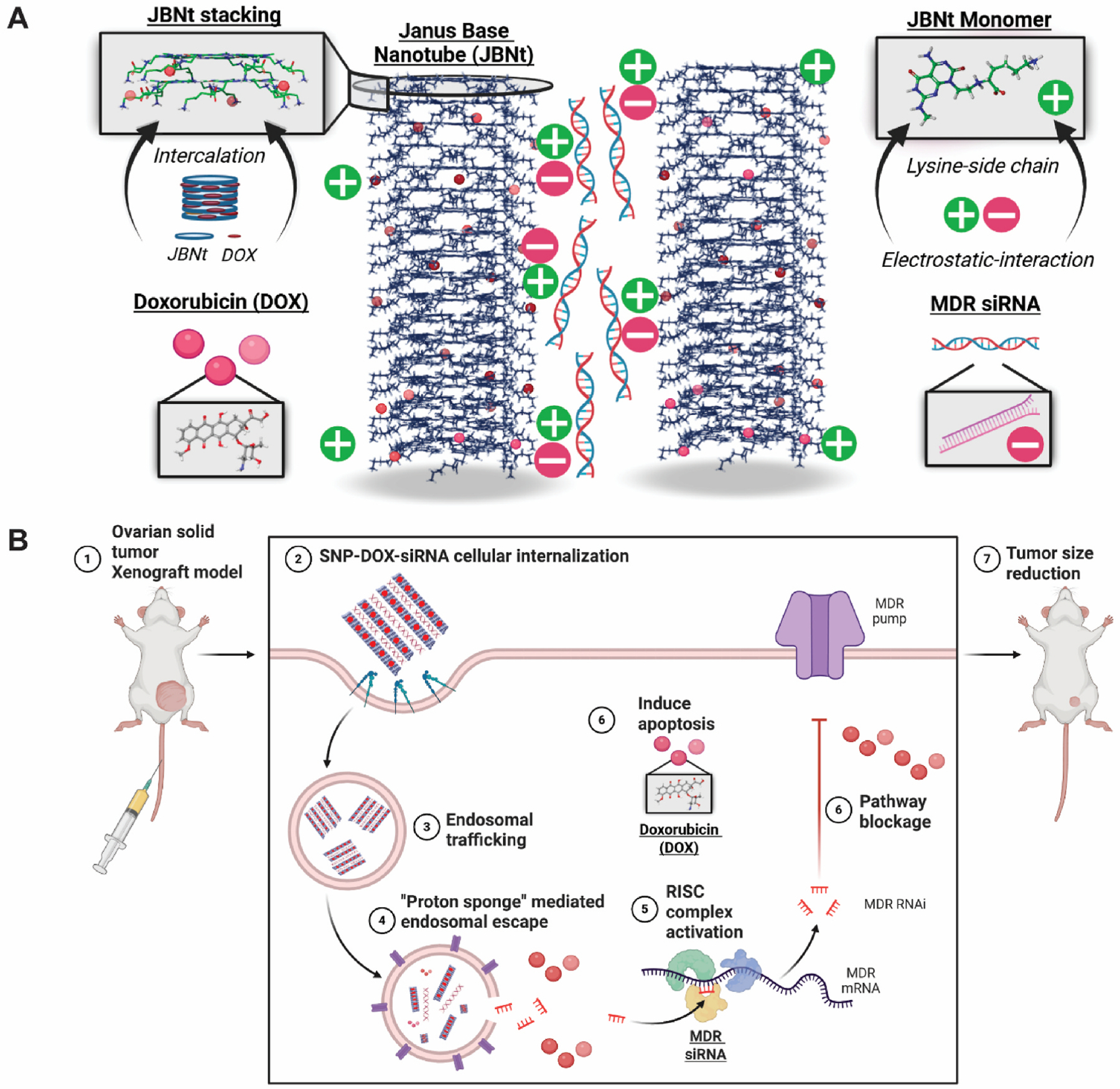
Schematic illustration of SNPs for the co-delivery of small molecules and siRNA for the treatment of solid tumors. (A) Self-assembly of SNP–DOX–siRNA through intercalation of doxorubicin (DOX) and electrostatic complexation of siRNA with Janus base nanotubes (JBNt). (B) Endosomal escape and intracellular delivery of SNP–DOX–MDR1 siRNA in an ovarian solid tumor xenograft mouse model.

We previously demonstrated that Janus base nanotubes (JBNt), DNA-mimicking nanostructures composed of self-assembling nucleobase units, enhance siRNA delivery through proton-sponge–mediated endosomal escape with minimal cytotoxicity [8,9]. However, these earlier formulations were not designed to support coordinated intracellular delivery of small-molecule therapeutics, where differences in subcellular trafficking and release kinetics often limit combination efficacy [10]. In the current study, we developed a sphere-like nanoparticle (SNP) from JBNt to enable dual-cargo co-delivery of siRNA and small-molecule drugs, leveraging the endosomal escape capability of JBNt within a single nanocarrier. In this system, doxorubicin (DOX) is intercalated within Janus base monomers via π–π stacking interactions, while siRNA is encapsulated through electrostatic interactions, enabling stable co-packaging (SNP-DOX-siRNA) (Fig. 1). Following cellular internalization, progressive endosomal acidification (pH ~6.0–5.5) promotes protonation-driven destabilization of DOX–JBNt interactions, expected to facilitate DOX release during early stages of endosomal maturation [11]. In parallel, siRNA delivery is preserved through proton-sponge–mediated escape from late endosomes, enabling efficient cytosolic access [8]. Together, this sequential yet complementary release mechanism enables spatially and temporally coordinated delivery of both therapeutic cargos, addressing a key limitation of conventional chemo–siRNA co-delivery platforms.

To validate this platform, we selected MDR1-mediated chemoresistance as a proof-of-concept resistance model, as MDR1 represents one of the most significant barriers to durable chemotherapeutic responses and frequently arises from mechanisms that cannot be addressed by single-agent therapies [12]. MDR1 is driven by coordinated cellular adaptations, including enhanced drug efflux, altered intracellular trafficking, and activation of pro-survival signaling pathways [13]. P-glycoprotein (P-gp), encoded by MDR1 (ABCB1), serves as the primary efflux transporter responsible for anthracycline resistance, making co-delivery of MDR1-targeting siRNA and DOX a rational strategy to simultaneously suppress P-gp expression and restore intracellular drug accumulation [14]. As a proof of concept, we demonstrate that SNP-mediated co-delivery, enabled by JBNt’s endosomal escape mechanism, achieves effective gene silencing and enhances apoptosis in cancer cells, tumor spheroids, and ovarian tumor xenograft models. These findings support JBNt-derived SNPs as a proof-of-concept platform for coordinated delivery of chemotherapeutics and siRNA in drug-resistant tumor models.

## 2. Results and discussion

### 2.1. Synthesis and characterization of SNP-DOX-siRNA

**Fig. 2.**
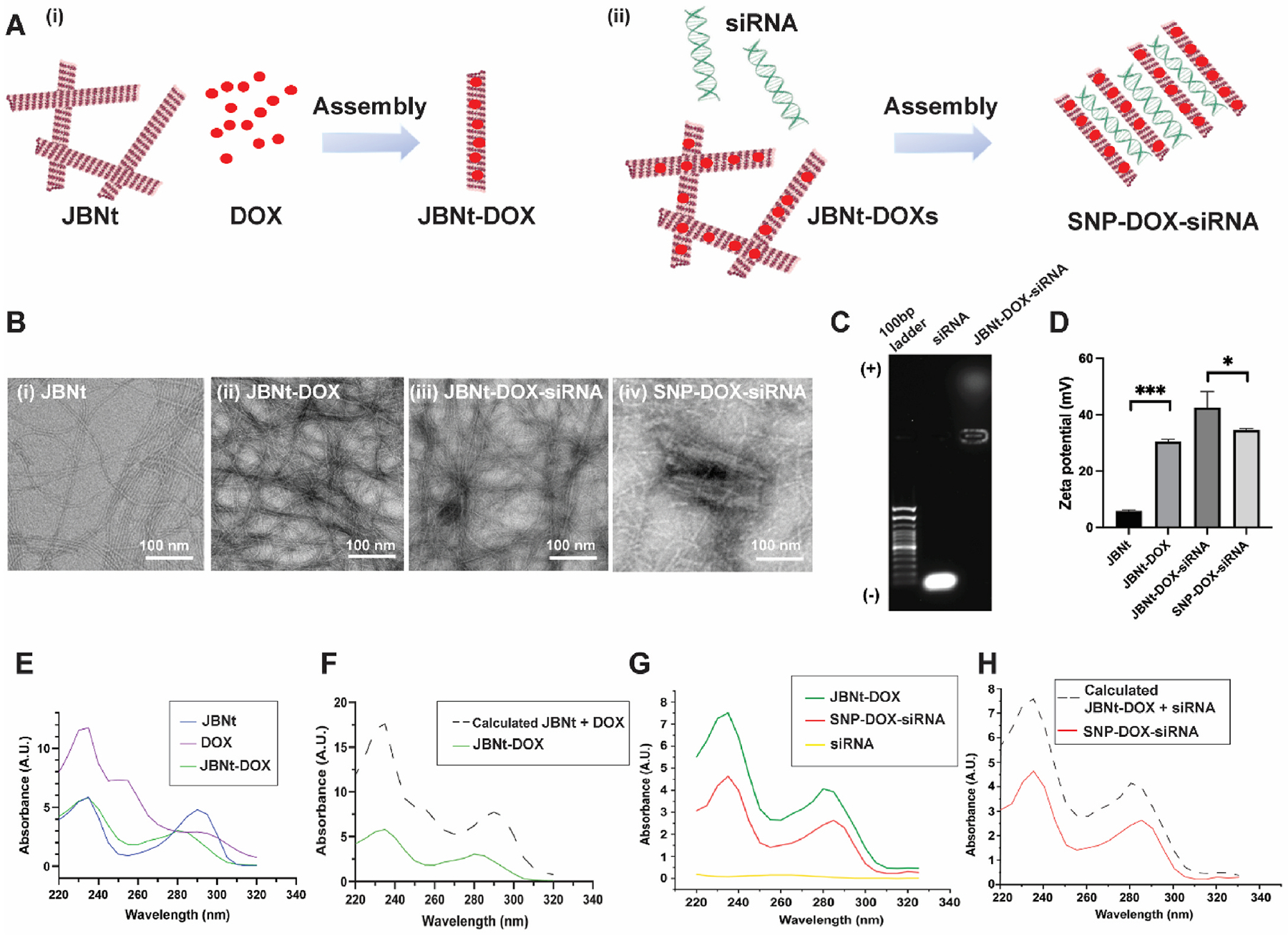
Synthesis and characterization of SNP-DOX-siRNA. (A) Intercalation of DOX into JBNt (i) and electrostatic interaction of siRNA with JBNt–DOX, leading to the self-assembly of SNP–DOX–siRNA (ii). (B) TEM images of (i) Janus base nanotube (JBNt), (ii) DOX-intercalted DOX (JBNt-DOX), (iii) JBNt-DOX with loaded siRNAs (JBNt-DOX-siRNA), and (iv) SNP loaded with DOX and siRNA (SNP-DOX-siRNA). (C) Agarose gel electrophoresis assay showing siRNA encapsulation at SNP-DOX-siRNA. (D) Zeta potential analysis (n = 3). (E) UV-Vis absorbance of JBNt-DOX. (F) UV-Vis analysis of JBNt-DOX. (G) UV-Vis absorbance of SNP-DOX-siRNA. (H) UV-Vis analysis of SNP-DOX-siRNA. Values reported are mean ± SEM. *P < 0.05, **P < 0.01, ***P < 0.001.

JBNt intercalated with doxorubicin (JBNt-DOX) self-assembled into a well-defined nanotubular structure as described in our previously published method [11]. Subsequently, siRNA was efficiently incorporated into JBNt-DOX through simple mixing driven by electrostatic interactions (Fig. 2A). TEM confirmed that JBNt-DOX retained its nanotubular morphology following DOX intercalation, and that subsequent siRNA complexation (JBNt-DOX-siRNA) similarly preserved this tubular structure (Fig. 2B). Upon sonication-assisted processing, JBNt-DOX-siRNA self-assembled into sphere-like nanoparticles (SNP-DOX-siRNA). The gel shift assay confirmed electrostatic complexation between siRNA and JBNt-DOX (Fig. 2C). JBNt-DOX successfully hindered the migration of siRNA in lane 3, whereas in lane 2, siRNA migrated easily toward the positive end of the gel. The ζ-potential measurements revealed significant differences between JBNt alone and JBNt-DOX (Fig. 2D). Surface charge decreased significantly between JBNt–DOX and siRNA mixture and SNP–DOX–siRNA, suggesting nanoparticle formation after sonication. The overall surface charge of SNP–DOX–siRNA was approximately +35 mV. To optimize SNP-DOX-siRNA formulation, the molar ratio of positively charged amine (N) groups in JBNt to negatively charged phosphate (P) groups in siRNA (N/P ratio) was systematically varied. Increasing the N/P ratio resulted in a highly positively charged surface potential and hydrodynamic diameters in the range of 200–300 nm (Fig. S1). The UV–Vis spectra further elucidated the interactions among the components of this formulation. In Fig. 2E, DOX exhibited a much higher absorbance than JBNt at 230 nm, and upon combination, JBNt–DOX showed a decreased absorbance along with a distinct spectral shift, particularly around 295 nm. This hypsochromic (blue) shift suggested successful drug intercalation into the JBNt structure. The difference is more clearly illustrated in Fig. 2F, which shows the calculated absorbance obtained by summing the absorbance values of DOX alone and JBNt alone. This calculated spectrum represented the expected outcome if the two molecules were present in solution without interaction. However, the experimentally observed spectrum (green) deviated markedly from this calculated profile, consistent with intercalation between JBNt and DOX upon combination. Fig. 2G and H showed a similar trend for siRNA, indicating electrostatic interactions that resulted in altered absorbance profiles. We further evaluated the stability and siRNA retention of the SNP-DOX-siRNA formulation over a 3-day period. UV–Vis spectroscopy demonstrated preservation of the characteristic absorbance profiles of SNP-DOX-siRNA throughout the study period, indicating structural stability of the nanoparticle formulation (Fig. S3). RiboGreen further analysis demonstrated high siRNA encapsulation efficiency and sustained retention, with more than 80% of siRNA remaining encapsulated with the nanoparticles after 3 days (Fig. S4).

### 2.2. In vitro co-delivery of DOX and siRNA

**Fig. 3.**
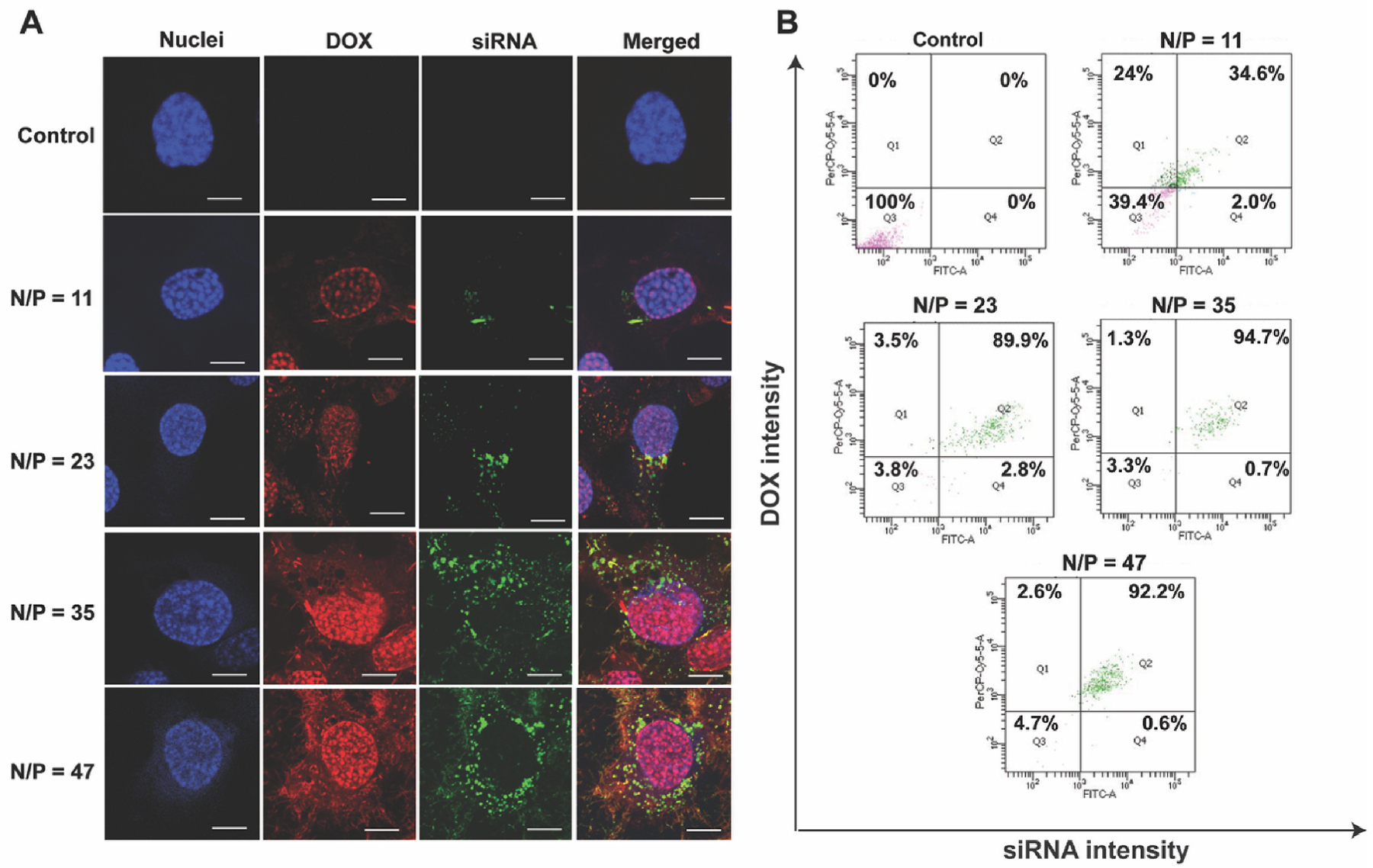
Optimization of the co-delivery of DOX and siRNA delivery via the SNP. (A) Co-delivery of the SNP-DOX-siRNA candidates: various JBNt-DOX ratios to AF488-siRNA delivered as SNP-DOX-siRNA to SKOV-3 cells for 24 h; images taken by CLSM. Scale bars are 10 *μ* m. (B) Co-delivery of the SNP-DOX-siRNA candidates; flow cytometry reported fluorescence intensity in SKOV-3 cells. Percentages are of the whole population sampled. Q2 indicates co-delivery.

We assessed co-delivery efficiency by varying N/P ratio, and treated human ovarian cancer cells (SKOV-3) with these formulations for 24 h, and analyzing intracellular distribution of DOX and AF488-tagged siRNA using confocal laser-scanning microscopy (CLSM). Across all tested N/P ratios, successful co-delivery of DOX (red) to the nucleus and siRNA (green) to the cytoplasm was observed (Fig. 3A). Quantitative analysis showed that an N/P ratio of 71 or higher provided effective siRNA uptake and DOX delivery in monolayer culture. Flow cytometry confirmed these results, demonstrating a high co-delivery efficiency of 92.2% at an N/P ratio of 47 (Fig. 3B). Based on these findings, we selected an N/P ratio of 47 for subsequent in vitro and in vivo cancer treatment studies.

### 2.3. SNP-DOX-siRNA exhibited superior endosomal escape compared with conventional LNP

**Fig. 4.**
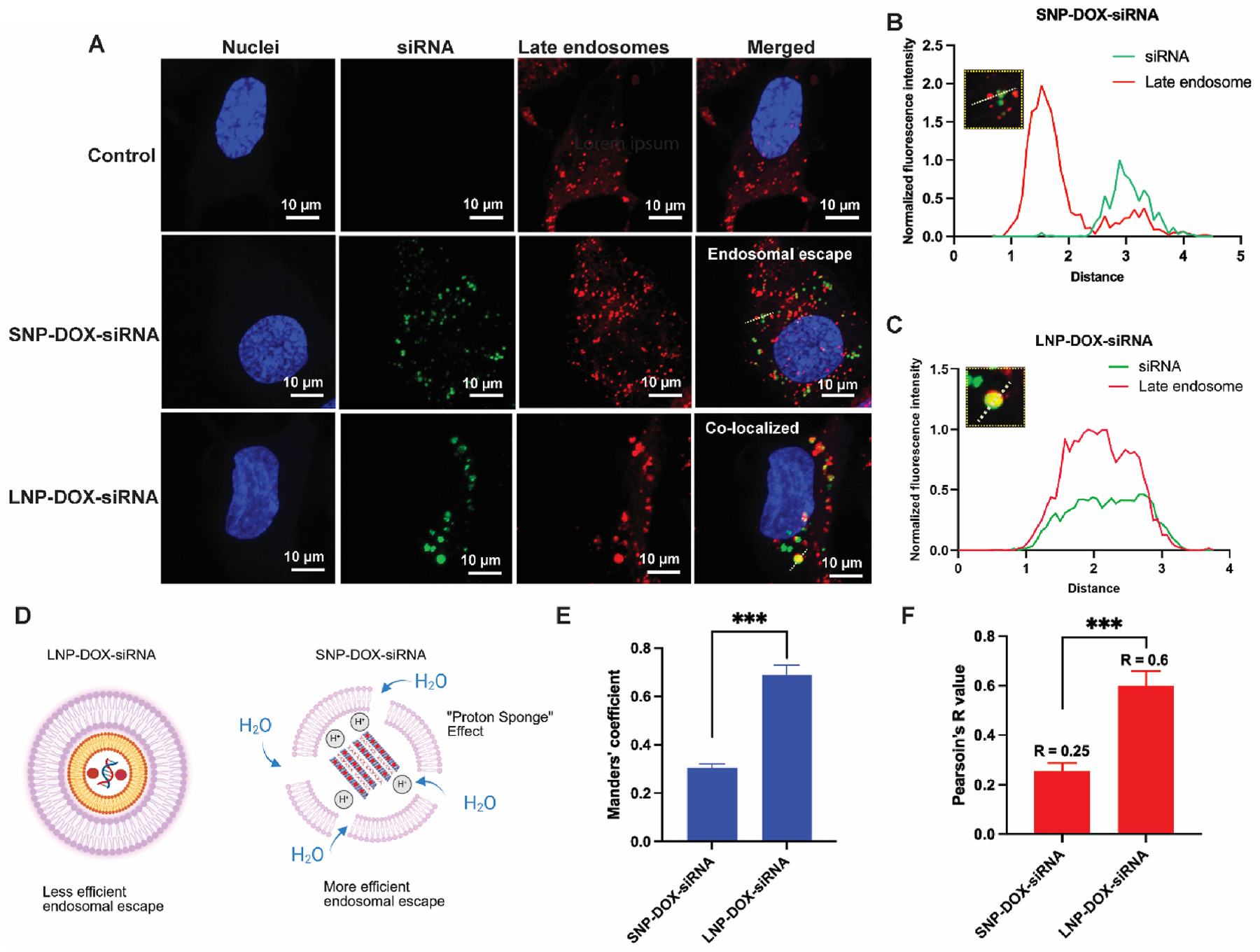
Endosomal escape of DOX and siRNA delivered by nanoparticles. (A) Confocal laser scanning microscopy (CLSM) images showing late endosomes (red), siRNA (green), and nuclei (blue) in cells treated with SNP-DOX-siRNA or LNP-DOX-siRNA. Nuclei were stained with DAPI. (B) Colocalization analysis of SNP-DOX-siRNA showing normalized fluorescence intensities of siRNA and late endosomes. (C) Colocalization analysis of LNP-DOX-siRNA showing normalized fluorescence intensities of siRNA and late endosomes. (D) Schematic illustrating that SNP–DOX–siRNA achieves more efficient endosomal escape via the proton sponge effect, leading to endosomal membrane rupture, whereas LNP–DOX–siRNA exhibits less efficient endosomal escape. (E) Manders’ coefficient calculated using ImageJ to quantify the degree of siRNA colocalization with late endosomes. (F) Quantification of siRNA colocalization with late endosomes expressed as Pearson’s correlation coefficient (R value, n = 6). *P < 0.05, **P < 0.01, and ***P < 0.001.

To evaluate SNP-mediated endosomal escape and intracellular delivery of siRNA and DOX, we conducted CLSM imaging with LysoTracker to label late endosomes and AF488-tagged siRNA co-delivered with DOX. Confocal imaging showed that SKOV-3 cells treated with SNP–DOX–siRNA exhibited bright, well-dispersed green fluorescence throughout the cytoplasm that did not co-localize with LysoTracker Red, suggesting that most of the siRNA successfully escaped from endosomal compartments (Fig. 4A). In comparison, the LNP–DOX-siRNA group displayed strong overlap between green and red fluorescence, indicating that a large portion of siRNA remained trapped inside late endosomes and lysosomes, as supported by the normalized fluorescence intensity analysis (Fig. 4B and C). Quantitative image analysis supported these observations. The calculated Manders’ coefficient of colocalization demonstrated that SNP-DOX-siRNA facilitated significantly higher endosomal escape than LNP-DOX-siRNA (Fig. 4E). The Pearson’s R value for the SNP-DOX-siRNA treated cells was significantly lower than that of the LNP-DOX-siRNA group (Fig. 4F), confirming that SNP facilitated more effective release of siRNA into the cytoplasm, consistent with proton-sponge buffering by JBNt nucleobase amine groups [8]. Overall, these results demonstrate that SNP significantly improves endosomal escape and the co-delivery of siRNA and DOX compared with conventional LNPs.

### 2.4. Co-delivery of DOX and siRNA enhanced apoptotic induction

**Fig. 5.**
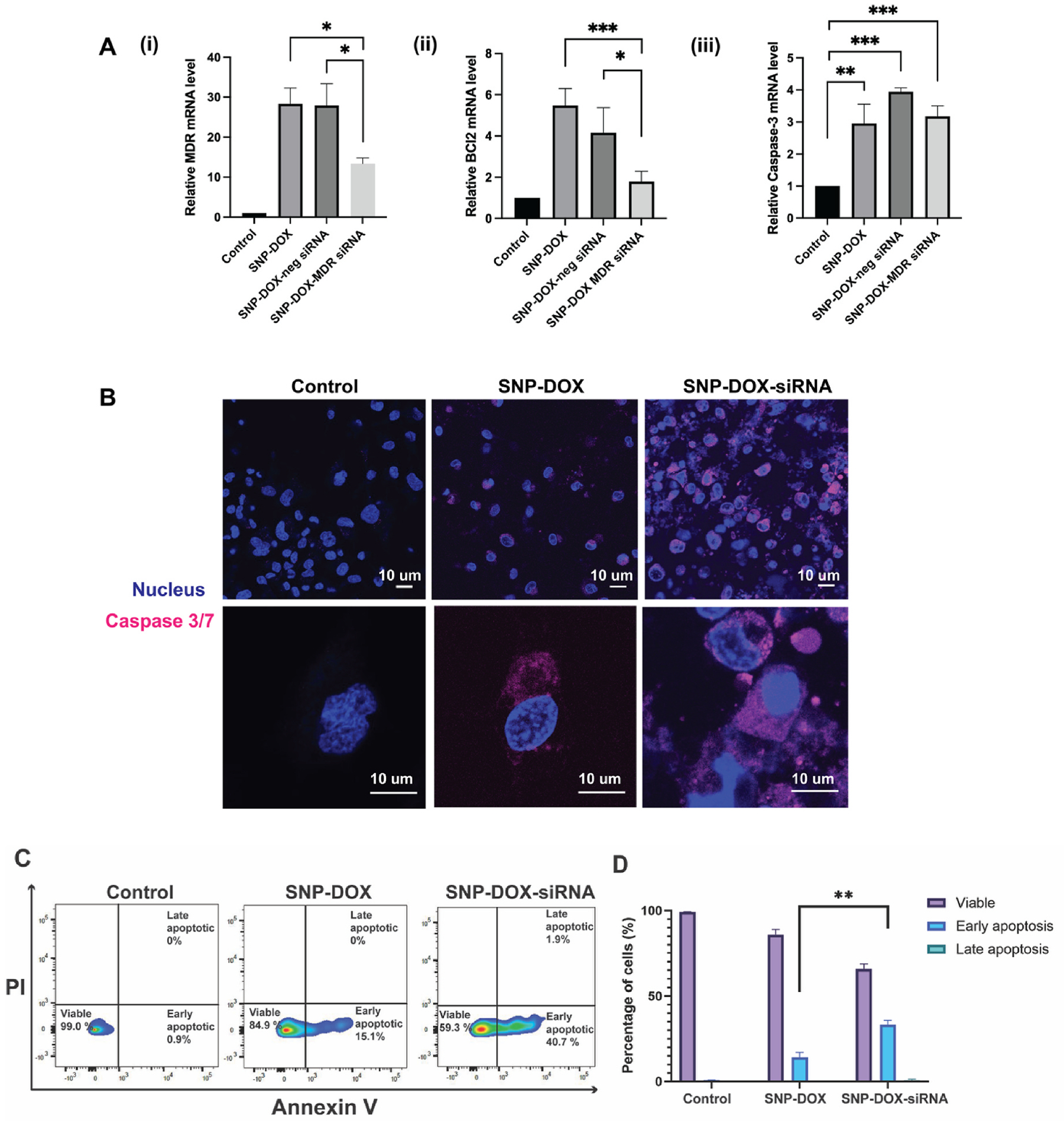
Functionality of MDR1 siRNA and DOX after co-delivery via SNP. (A) RT-qPCR analysis of (i) MDR1, (ii) BCL-2, and (iii) Caspase-3 mRNA expression 24 h after treatment with SNP-DOX, SNP-DOX-MDR1 siRNA, SNP-DOX-neg siRNA or negative control (n = 3). (B) Caspase-3/7 staining of SKOV-3 cells after 1 day of transfection with SNP-DOX, SNP-DOX-siRNA, or a negative control; images taken by CLSM. (C) Annexin V apoptosis was evaluated 24 h after treatment with SNP-DOX, SNP-DOX-MDR1 siRNA, or a negative control. Apoptotic cell populations were quantified by flow cytometry. (D) Quantification of apoptosis. Percentages of viable, early apoptotic, and late apoptotic cell populations (n = 3). Scale bars are 10 *μ* m. Values reported are mean ± SEM. *P < 0.05, **P < 0.01, ***P < 0.001.

To evaluate whether SNP-DOX-siRNA achieves coordinated biological function through gene silencing and downstream apoptotic signaling, we performed qPCR to quantify mRNA expression. MDR1 mRNA expression was significantly reduced in the SNP-DOX-MDR1 siRNA group compared with the SNP-DOX and SNP-DOX-neg siRNA (scrambled negative-control siRNA) groups, which lacked MDR1-targeting siRNA (Fig. 5A). To further validate MDR1 silencing at the protein level, we performed western blotting and flow cytometric analysis using untreated control, SNP-DOX, SNP-DOX-negative control siRNA, and SNP-DOX-MDR1 siRNA groups. Western blotting showed reduced P-gp expression following SNP-DOX-MDR1 siRNA treatment (Fig. S7), and flow cytometry further showed a significant decrease in MDR/P-gp signal compared with the SNP-DOX-negative control siRNA group (Fig. S8). Together with MDR1 mRNA knockdown and enhanced apoptotic induction, these results support MDR1/P-gp suppression as a contributing mechanism to the improved chemotherapeutic efficacy of SNP-DOX-MDR1 siRNA beyond DOX cytotoxicity alone. This gene silencing was accompanied by upregulation of pro-apoptotic caspase-3 mRNA in both SNP-DOX and SNP-DOX-siRNA groups, and a concomitant decrease in anti-apoptotic BCL-2 expression in the SNP-DOX-siRNA group relative to SNP-DOX and SNP-DOX-neg siRNA groups. Consistent with these transcriptional changes, caspase-3/7 staining revealed markedly elevated apoptotic activity in SNP-DOX-siRNA-treated cells relative to SNP-DOX controls (Fig. 5B). Annexin V apoptosis assays corroborated these molecular findings (Fig. 5C). The SNP-DOX-siRNA group showed a significant increase in the percentage of cells in early apoptosis, with 40.7% of cells as compared to 15.1% in SNP-DOX treated cells (Fig. 5D).

### 2.5. Co-delivery of DOX and siRNA enhanced penetration and apoptosis in cancer spheroids

**Fig. 6.**
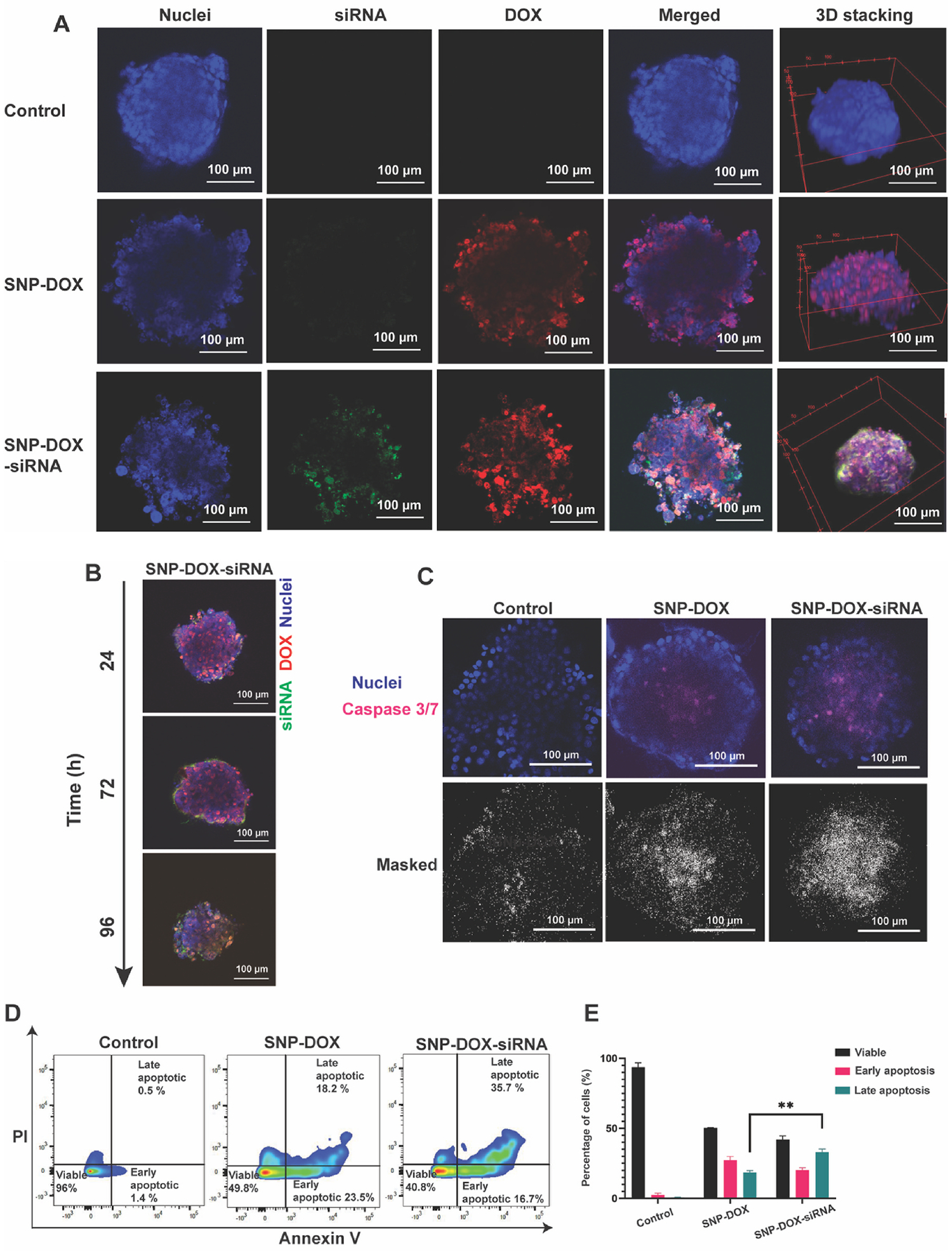
Co-delivery of DOX and siRNA to ovarian cancer spheroids (SKOV-3 spheroids) via the SNPs. (A) Delivery of SNP-DOX and SNP-DOX-siRNA to SKOV-3 spheroids for 4 days; images taken by CLSM. (B) Time-dependent delivery of SNP-DOX-siRNA to SKOV-3 spheroids over 4 days. Timepoint images taken at 24h, 72h, and 96h post-transfection; images taken by CLSM. (C) Caspase-3/7 staining of SKOV-3 spheroids after 3 days of transfection with SNP-DOX, SNP-DOX-siRNA, or a negative control; images taken by CLSM. (D) Annexin V apoptosis assay results after 3 days of delivery with SNP-DOX, SNP-DOX-siRNA, or negative control to SKOV-3 spheroids. Apoptosis was analyzed using flow cytometry. (E) Quantification of apoptosis (n = 3). Values reported are mean ± SEM. *P < 0.05, **P < 0.01, ***P < 0.001. Scale bars are 100 *μ* m.

We next assessed the in vitro delivery of SNP-DOX-siRNA to ovarian cancer spheroids (SKOV-3 spheroids). SNP-DOX effectively delivered DOX throughout SKOV-3 spheroids, while SNP-DOX-siRNA achieved co-delivery of both siRNA and DOX into the spheroid core (Fig. 6A). In a time-dependent study, SNP-DOX-siRNA demonstrated time-dependent penetration, with delivery depth increasing progressively from 24 to 96 h, achieving distribution throughout the spheroid core by the 96-h time point (Fig. 6B). Similar to the monolayer culture, we delivered SNP-DOX-MDR1 siRNA to the cancer spheroids and observed that the apoptosis assays showed increased Caspase-3/7 activity in the SKOV-3 spheroids. Through Caspase-3/7 staining, a high number of cells exhibited Caspase-3/7 expression deep within spheroid cores in the SNP-DOX-siRNA treated group (Fig. 6C). Notably, while SNP-DOX alone induced caspase-3/7 activation primarily at the spheroid periphery, the addition of MDR1-siRNA co-delivery via SNP-DOX-siRNA substantially enhanced apoptotic induction throughout the entire spheroid volume, including the core. To quantify apoptosis, we performed the Annexin V assay in the SKOV-3 spheroids (Fig. 6D). The SNP-DOX-siRNA group showed a much higher percentage of cells in the late apoptosis stage, with a population of 35.7% as compared to 18.2% in SNP-DOX treated cells (Fig. 6E).

### 2.6. Antitumor efficacy of SNP-mediated co-delivery of DOX and siRNA

**Fig. 7.**
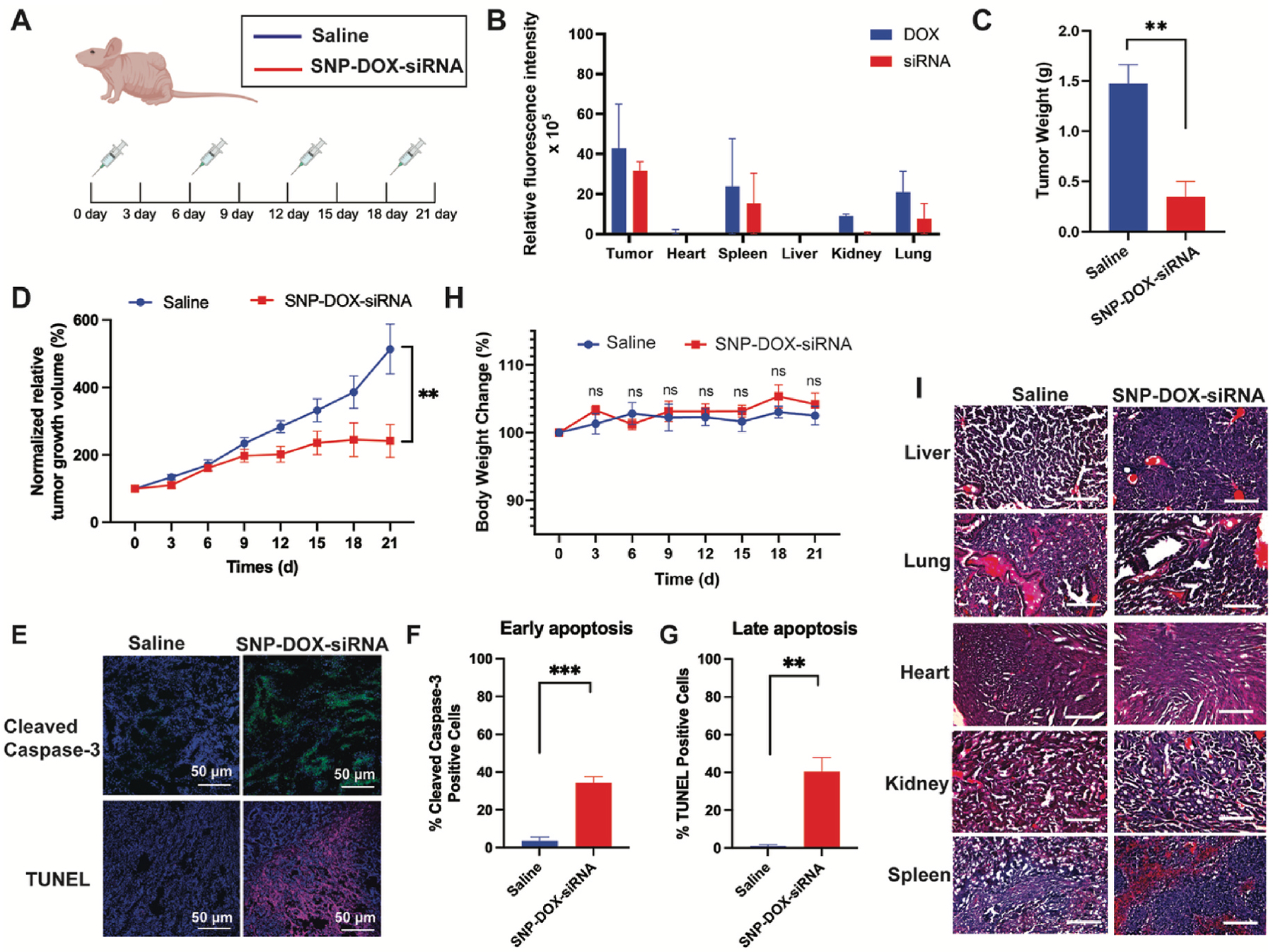
Co-delivery of DOX and MDR1 siRNA via SNP to treat SKOV-3 xenograft tumor mice model. (A) Schematic illustration of the treatment plan. (B) ROI-based ex vivo quantification of DOX and AF647-tagged siRNA fluorescence signals in tumor tissue and major organs, including heart, spleen, liver, kidney, and lung, 3 days after intravenous administration of SNP-DOX-siRNA (n = 3). Fluorescence intensity was background-subtracted and expressed as relative fluorescence intensity for each tissue. (C) Weight of excised tumor (n = 6). (D) Relative tumor-growth curves during the treatment were measured by a caliper (n = 6). (E) Immunofluorescence staining of cleaved caspase-3 (middle), and TUNEL analysis (bottom) of the tumors, scale bar: 100 μm. (F) Quantification of cleaved caspase-3 positive cells. (G) Quantitation of apoptotic cells from TUNEL staining (n = 6). (H) Body weight change of mice (n = 6). (I) Major organs were obtained for H&E staining. Scale bar = 100 μm. Values reported are mean ± SEM. *P < 0.05, **P < 0.01, ***P < 0.001.

To translate our in vitro findings toward therapeutic application, we evaluated the in vivo antitumor efficacy of SNP-DOX-siRNA in the SKOV-3 tumor-bearing nude mouse model (Fig. 7A). To assess tumor accumulation and organ distribution of SNP-DOX-siRNA, we performed ex vivo fluorescence imaging 3 days after intravenous administration. Major organs and tumors were collected, and the fluorescence signals of DOX and AF647-labeled siRNA were quantified in the tumor, heart, spleen, liver, kidney, and lung (Fig. 7B). Representative IVIS images are shown in Fig. S10. SNP–DOX–siRNA exhibited enhanced accumulation of both DOX and siRNA in tumors compared to major organs, although substantial accumulation was also observed in the spleen and lung. Based on this observed tumor accumulation, we evaluated the therapeutic efficacy of SNP-DOX-siRNA in the same SKOV-3 tumor model. Once tumors reached approximately 200 mm^3^, mice were randomized into two groups (saline control and SNP-DOX-siRNA treatment). Treatments were administered via intravenous injection every six days over a 21-day period. Tumor volume and body weight were monitored throughout the study to evaluate therapeutic response and systemic tolerability. The final tumor weights were markedly lower in the SNP–DOX–siRNA group (Fig. 7C). In addition, as shown in Fig. 7D, tumors in the saline group grew rapidly, whereas tumor growth in the SNP–DOX–siRNA–treated group was significantly inhibited over the 21-day treatment period, consistent with the antitumor efficacy of SNP-DOX-siRNA. Immunofluorescence analysis of tumor cryosections demonstrated strong activation of apoptotic markers in the treatment group (Fig. 7E). Tumors from SNP–DOX–siRNA–treated mice exhibited intense cleaved caspase-3 (Fig. 7F) and TUNEL signals (Fig. 7G), whereas saline-treated controls showed minimal fluorescence, indicating extensive apoptosis induction consistent with the observed tumor regression. No apparent systemic toxicity was observed. Mice maintained stable body weight throughout the study (Fig. 7H). Histological examination of major organs (liver, lung, heart, kidney, and spleen) revealed normal tissue morphology comparable to the saline group, with no evidence of necrosis or inflammation (Fig. 7I). Moreover, blood was collected for a complete blood count (CBC), which included white blood cell (WBC), red blood cell (RBC), hemoglobin (HGB), platelet (PLT), neutrophil (NEU), lymphocyte (LYM), monocyte (MON), and hematocrit (HCT) analyses. Comparisons were made relative to the pre-injection baseline for mice in each group. Differential CBC analysis revealed that the SNP-DOX-siRNA did not induce changes in the levels of any of the above-mentioned blood components (Fig. S11). Together, these results demonstrate that SNP–DOX–MDR1 siRNA achieves significant tumor growth inhibition mediated through coordinated delivery of DOX and MDR1 siRNA, while maintaining systemic biocompatibility as evidenced by stable body weight and normal organ histology.

## 3. Discussion

Endosomal sequestration is a major obstacle to effective siRNA delivery in nanoparticle-based co-delivery systems [5,15]. Despite the development of platforms such as LNPs and polymeric nanoparticles for co-loading doxorubicin and siRNA, therapeutic efficacy is constrained by inefficient endosomal escape of the RNA payload [16–18]. After cellular uptake, siRNAs trapped within endosomal compartments are subjected to acidification and degradative enzymes, causing suboptimal gene silencing efficacy [19]. Quantitative analyses indicate that fewer than 2% of siRNA molecules delivered by conventional LNPs reach the cytosol, with most undergoing lysosomal degradation or extracellular recycling [20,21]. This challenge is intensified in MDR1 cancer cells, which often display increased endo-lysosomal trafficking and active sequestration or extrusion of therapeutic agents, further diminishing cytosolic siRNA delivery [22]. The SNP platform, built upon JBNt technology, was designed to address the endosomal escape limitation of RNA delivery. In the present study, reduced siRNA colocalization with LysoTracker-labeled endosomal/lysosomal compartments was observed for SNP-DOX-siRNA relative to the LNP, which is consistent with enhanced endosomal escape. However, the present colocalization analysis does not directly establish the mechanism responsible for this observation. Previous studies of JBNt-derived nanomaterials have reported proton-sponge-associated buffering behavior linked to enhanced endosomal escape [8]. Therefore, the enhanced endosomal escape observed in the present study could potentially involve JBNt-associated proton-sponge effects, although this remains hypothetical and was not directly demonstrated in the current SNP-DOX-siRNA formulation. Future studies will perform additional mechanistic investigations, including bafilomycin A1 or chloroquine inhibition, endosomal pH perturbation analysis, and galectin-8 recruitment imaging [23–25].

This co-delivery platform is especially pertinent for drug-resistant tumors such as ovarian adenocarcinoma, where MDR1 significantly restricts intracellular drug accumulation and therapeutic efficacy [13,26]. Doxorubicin monotherapy is frequently inadequate due to P-gp-mediated efflux, which rapidly removes the drug from cancer cells and reduces intracellular retention by up to tenfold compared to drug-sensitive cells [27]. Incorporation of siRNA targeting MDR1 enables concurrent cytotoxic treatment and genetic suppression of the primary resistance mechanism [28]. This combinatorial approach directly addresses the mechanistic basis of MDR1 and provides a rational strategy to restore drug sensitivity in resistant cancer cells. Previous studies have demonstrated that siRNA-mediated P-gp silencing can re-sensitize MDR1 cell lines to chemotherapy [17,29,30]. Although this represents a substantial therapeutic advance, antitumor efficacy may be further improved by employing alternative RNA cargos that target additional resistance or survival pathways. For instance, mRNA encoding pro-apoptotic proteins [31], tumor-suppressor miRNAs such as miR-34a [32], and circular RNAs targeting survival signaling pathways [33] offer considerable potential to enhance chemotherapeutic outcomes beyond P-glycoprotein silencing. Future research should systematically evaluate the co-delivery of diverse functional RNAs with chemotherapeutics to optimize antitumor responses and broaden the applicability of combinatorial solid tumor therapy.

In addition to target selection, the physicochemical properties of the delivery vehicle play a critical role in determining therapeutic outcomes. The dual-loading strategy employed in this study, which involves sequential intercalation of doxorubicin followed by electrostatic complexation with siRNA, enables stable co-packaging of both agents within a single nanocarrier and eliminates the need for separate nanocarriers to deliver agents. This unified carrier approach offers significant advantages over two-component delivery systems, in which differences in pharmacokinetics can result in asynchronous delivery of the drug and RNA, thereby potentially reducing the synergistic effects of simultaneous gene silencing and cytotoxic activity [34,35].

The shape of nanoparticles may influence their intratumoral transport. In three-dimensional SKOV-3 spheroids, SNP–DOX–siRNA progressively penetrated beyond the periphery and reached the spheroid core, indicating effective diffusion through a dense tumor-like structure. In our previous study, rod-shaped JBNt with aspect ratios (the length divided by the width) greater than 5.3 demonstrated enhanced tumor infiltration compared to spherical particles, attributed to improved interstitial transport [11]. Although the present SNP exhibits a sphere-like morphology with a lower aspect ratio (~1.6), these findings suggest that nanoparticle shape remains an important and tunable design parameter within the JBN platform (Fig. S2). Future optimization toward higher aspect ratios may further improve intratumoral penetration and therapeutic distribution in solid tumors. In addition to morphology optimization, the modular chemistry of JBNt allows for extensive platform customization. JBNt side chains can be functionalized with targeting ligands, such as folate [36], transferrin [37], or tumor-homing peptides, such as iRGD [38], to enhance selective accumulation in tumors that express the corresponding receptors. This modular surface modification supports the creation of a JBN library with varied side-chain chemistries, which can be systematically evaluated in future studies to optimize tumor-targeting and delivery efficacy.

Several limitations of the current study should be acknowledged. First, in vivo efficacy was evaluated only in the SKOV-3 ovarian cancer xenograft model, which limits assessment of platform generalizability. Future validation in additional multidrug-resistant models is feasible because the SNP platform is modular: DOX loading is mediated by the JBNt, while the siRNA cargo can be adapted to the dominant resistance pathway in each tumor model. For example, SNP-DOX-siRNA could be evaluated in ABCB1/P-gp-associated breast cancer models such as MCF-7/ADR [39] and doxorubicin-resistant lung cancer models such as A549/DOX [40]. Such studies will help determine whether this co-delivery strategy is broadly applicable beyond the SKOV-3 ovarian cancer model [13,41]. Second, the in vivo efficacy study included only saline and SNP-DOX-MDR1 siRNA treatment groups. Thus, the current data provide proof-of-concept evidence of antitumor activity but do not fully distinguish the individual contributions of DOX, MDR1 siRNA, and the SNP carrier, nor do they establish in vivo synergy. Future studies will include more comprehensive experimental designs with time- and dose-ranging studies and expanded control groups, including free DOX, SNP-DOX, SNP-DOX-NC siRNA, free DOX plus MDR1 siRNA, and SNP-DOX-MDR1 siRNA. Third, the lipid-based comparator, LNP, used here was a Lipofectamine 2000 formulation, a widely used cationic lipid transfection reagent [42]. Although Lipofectamine provides a well-established reference for endosomal escape comparison, comparison with clinically relevant ionizable LNP platforms in future studies would provide a more appropriate translational context for evaluating the endosomal escape advantage conferred by JBNt [43,44]. Fourth, while the present data support enhanced tumor accumulation and therapeutic efficacy, comprehensive pharmacokinetic and biodistribution profiling, including clearance kinetics, organ-level accumulation, and immunogenicity assessment, remains to be performed as part of preclinical development [45]. Finally, long-term toxicity assessment beyond the 21-day treatment window was not conducted in this study; extended dosing and chronic safety evaluation will be essential as this platform advances toward clinical translation [46]. Despite these limitations, the current findings provide proof-of-concept evidence for JBNt-derived SNPs as a co-delivery platform and establish a foundation for continued optimization.

In summary, this study establishes SNP, a nanocarrier developed from JBNt, as a co-delivery platform that addresses a central limitation of conventional nanocarriers: inefficient endosomal escape of RNA therapeutics. SNP-DOX-siRNA showed significantly reduced endosomal/lysosomal siRNA colocalization compared with the LNP, supporting enhanced cytosolic delivery of siRNA and coordinated intracellular delivery of doxorubicin. Collectively, these results highlight the potential of SNP-based co-delivery systems leveraging JBNt endosomal escape technology for combinatorial RNA chemotherapy in drug-resistant solid tumors.

## 4. Materials and methods

### 4.1. Materials

JBNt monomer was synthesized according to previously reported procedures and purified using high-performance liquid chromatography (HPLC) [47–49]. DOX free base was purchased from MedKoo Biosciences. Tert-butanol (t-BuOH) was purchased from Alfa Aesar. SKOV-3 cells were obtained from ATCC. McCoy’s 5A cell culture medium (Gibco), Trypsin-EDTA (0.25%, Gibco), fetal bovine serum (FBS, Gibco), phosphate-buffered saline (PBS, Gibco), ethanol (70% solution), and Lipofectamine 2000 were obtained from Thermo Fisher Scientific. Triton X-100 (1.0%, Invitrogen), DAPI, and 4% formaldehyde (Invitrogen) were purchased from Fisher Scientific. 24-well and 96-well plates (Corning) were purchased from Fisher Scientific.

### 4.2. Methods

#### 4.2.1. Fabrication of SNP-DOX-siRNAs

Lysine-side-chain JBNt was mixed with DOX. The final concentration of DOX was 20 *μ* g/mL, and the final concentration of JBNt was 1 mg/mL. JBNt-DOX was stored at 4 °C for two weeks for further self-assembly. JBNt-DOX was mixed with AllStars negative control siRNA (Qiagen), AllStars AF488-tagged siRNA (Qiagen), siRNA at varying N/P ratios under sterile conditions, followed by sonication (Q Sonica; Sonicator) at 100% amplitude for 2 min and 30 s. The resulting nanoparticle was termed SNP-DOX-siRNA.

#### 4.2.2. Characterization of SNPs

The morphology of JBNt-DOX was observed by transmission electron microscopy (TEM; Tecnai T12). The hydrodynamic size and zeta potential of JBNt were measured by dynamic light scattering (Zetasizer, Malvern Panalytical) after dilution with Milli-Q water. A gel shift assay was used to evaluate siRNA encapsulation by JBNt-DOX using a 0.8% agarose gel prepared from UltraPure agarose in TBE buffer and supplemented with SYBR Safe DNA gel stain (Thermo Fisher Scientific) for nucleic acid visualization. Samples were mixed with 10× BlueJuice Gel Loading Buffer (Thermo Fisher Scientific) to facilitate loading and tracking during electrophoresis. A 100 bp DNA ladder (Invitrogen) was included to aid sample visualization. The gel was run at 100 V for 50 min and imaged using an iBright 1500 system (Invitrogen). UV–visible (UV–Vis) absorption spectra were acquired using a NanoDrop One/OneC spectrophotometer over a wavelength range of 180–350 nm. To assess the stability of SNP-DOX-siRNA, UV–Vis absorbance spectra were recorded over a 3-day period. Nanoparticle samples were stored at room temperature in nuclease free water and measurements were taken at predetermined time points (0–3 days). The siRNA encapsulation efficiency of SNP-DOX-siRNA nanoparticles was quantified using a RiboGreen RNA assay (Quant-iT^™^ RiboGreen Reagent, Thermo Fisher Scientific). Freshly prepared SNP-DOX-siRNA nanoparticles were incubated in nuclease-free water for 0–3 days. At each time point, aliquots were collected and mixed with diluted RiboGreen reagent according to the manufacturer’s protocol to quantify accessible or unencapsulated siRNA. Heparin was used as an anionic competitor to disrupt electrostatic interactions between siRNA and SNP-DOX, allowing quantification of total siRNA amount following siRNA release. Samples were then mixed with diluted RiboGreen reagent according to the manufacturer’s instructions, and fluorescence was measured at excitation/emission wavelengths of approximately 480/520 nm. siRNA concentrations were calculated from a free-siRNA standard curve after subtracting background fluorescence from SNP-DOX without siRNA, heparin-only, and buffer-only controls.

Encapsulation efficiency (EE%) was calculated as:

$$\text{EE}(\%)\frac{\text{Total siRNA}(\text{with heparin})\text{-unencapsulated siRNA}(\text{without heparin})}{\text{Total siRNA}(\text{with heparin})}\times 100\%,$$

and reported as mean ± SEM.

#### 4.2.3. Spheroid culture

SKOV-3 cells were seeded on 96-well Nunclon Sphera round-bottom plates (Thermo Fisher Scientific) at a density of 2 × 10^3^ cells/well. Cells were incubated for 5 days to achieve compact and consistent spheroids before subsequent experiments. Spheroid formation was monitored daily using a ZOE Fluorescent Cell Imager (Bio-Rad). Spheroid diameters were quantified from bright-field images using ImageJ to assess spheroid growth, compaction, and uniformity before treatment. After 5 days of culture, compact spheroids with an average diameter of 445.2 ± 10 μm were obtained and used for subsequent penetration, delivery, and apoptosis experiments (n = 8; Fig. S5).

#### 4.2.4. SNP-DOX-siRNA delivery to cells

SKOV-3 cells were seeded on Lab-Tek 8 well chambered coverglass (Thermo Fisher Scientific) at a density of 5 × 10^4^ cells/mL. After 24 h, SNP-DOX-siRNA formulations at different N/P ratios of JBNt-DOX/AF488-tagged siRNA were added to the corresponding wells, achieving a final DOX concentration of 8 μg/mL and a final siRNA concentration of 50 nM. A no-treatment control was included. Twenty-four hours later, cells were carefully washed with PBS and fixed with 4% formaldehyde for 15 min. The cells were carefully washed again and treated with 0.1% Triton X-100 for 10 min to increase the permeability of the cell membranes. After washing, DAPI was incubated for 5 min to stain nuclei. The cells were washed the final two times with PBS and protected from light until imaged. Cells were fluorescently imaged using a Nikon A1 confocal laser-scanning microscope. Images were analyzed using ImageJ software. SKOV-3 spheroids (5 days, 37 °C and 5% CO_2_) were transfected with JBNt-DOX and SNP-DOX with AF488-siRNA and incubated for 4 days at 37 °C and 5% CO_2_. Spheroids were carefully washed with PBS twice, were incubated with 4% formaldehyde for 24 h in 4 °C, and then washed carefully with PBS twice. Hoechst 33342 (Thermo Fisher) was used to stain nuclei of single cells in spheroids, diluted to 8 *μ* M, and incubated for 24 h at 4 °C. The spheroids were washed a final two times with PBS, transferred to a Lab-Tek 4 well chambered coverglass (Thermo Fisher Scientific). Cells were fluorescently imaged using a Nikon A1 confocal laser-scanning microscope. Images were analyzed using ImageJ software. For the time dependent study, the steps explained above were completed after 24, 72, and 96 h of incubation with treatments. For spheroid penetration analysis, confocal z-stack images were collected from treated spheroids, and the central optical section was used for radial fluorescence intensity analysis. DOX and AF488-siRNA penetration were quantified using ImageJ by measuring fluorescence intensity profiles from the spheroid periphery toward the core. Penetration depth was defined as the distance from the spheroid surface at which the fluorescence signal remained above background intensity. Representative images from untreated control, SNP-DOX, and SNP-DOX-siRNA groups were included for comparison.

#### 4.2.5. Flow cytometry

For monolayer culture, SKOV-3 cells were seeded in 24-well plates (Fisher Scientific) and transfected for 24 h with SNP-DOX or SNP-DOX with AF488-tagged siRNA (SNP-DOX-siRNA), achieving a final DOX concentration of 8 μg/mL and a final siRNA concentration of 50 nM. A no-treatment negative control was included. To visualize colocalization of DOX and siRNA in SKOV-3 cells, flow cytometry was used. The cells were lifted using Trypsin/EDTA 0.25% (as described in the culture methods). After neutralizing with media, the cells were centrifuged at 1340 RPM for 4 min. Supernatant was carefully removed, and the cells were resuspended in PBS and centrifugation was repeated. The supernatant was carefully removed, and the cells were resuspended in PBS for flow cytometry. To determine SNP-DOX-siRNA delivery to SKOV-3 cells, flow cytometry was carried out using a BD LSRFortessa X-20 Cell Analyzer (BD Biosciences) using FITC-A and PerCP-Cy5-5-A lasers, and analysis was completed with BD FACSDiva software (Fig. S6). To assess MDR (P-gp) expression, SKOV-3 cells were seeded in 24-well plates and treated with untreated control, SNP-DOX, SNP-DOX-negative control siRNA, or SNP-DOX-MDR1 siRNA for 72 h. Cells were then washed with PBS, detached using trypsin-EDTA, collected by centrifugation, and resuspended in staining buffer. To evaluate P-gp protein expression, cells were fixed and permeabilized by using eBioscience^™^ Intracellular Fixation & Permeabilization Buffer Set (Thermofisher Scientific). Then, permeabilizaed cells were incubated with an anti-MDR/P-gp primary antibody for 30 min, followed by an AF488-conjugated secondary antibody for 1h. After washing, cells were resuspended in PBS and analyzed by flow cytometry using the FITC channel. The percentage of MDR/P-gp-positive cells and mean fluorescence intensity were quantified, and fold change was calculated relative to the untreated control.

#### 4.2.6. Western blot analysis

SKOV-3 cells were seeded in culture plates and allowed to attach overnight before treatment with untreated control, SNP-DOX, SNP-DOX-negative control siRNA, or SNP-DOX-MDR1 siRNA. Treatments were prepared using the same in vitro dosing conditions used for the functional assays, with a final DOX concentration of 8 μg/mL and siRNA concentration of 50 nM. After 72 h of treatment, cells were washed with cold PBS and lysed using RIPA buffer supplemented with protease inhibitor. Cell lysates were clarified by centrifugation, and total protein concentration was determined using a BCA protein assay. Equal amounts of protein were separated by SDS-PAGE and transferred onto PVDF membranes. Membranes were blocked with EveryBlot blocking buffer (Biorad) and incubated overnight at 4 °C with primary antibodies against MDR1/ABCb1 Rabbit monoclonal antibody (E1Y7B, Cell Signaling Technology) and rabbit anti β-actin(AHP2417, Bio-Rad). After washing, membranes were incubated with HRP-conjugated secondary antibodies, and protein bands were visualized using enhanced chemiluminescence. Band intensities were quantified using ImageJ, and P-gp expression was normalized to β-actin. Relative P-gp expression was calculated relative to the untreated control group. Data are presented as mean ± SEM from three independent experiments, and statistical significance was determined using one-way ANOVA followed by Tukey’s multiple-comparisons test, with *P* < 0.05 considered significant.

#### 4.2.7. Apoptosis assay

For the monolayer apoptosis assay, SKOV-3 cells were seeded at a density of 5 × 10^4^ cells/mL in 24-well plates. After 24 h of incubation, SNP-DOX or SNP-DOX with MDR1-siRNA (SNP-DOX-MDR1-siRNA) was added to the cells, achieving a final DOX concentration of 8 μg/mL and a final siRNA concentration of 50 nM, and incubated at 37 °C with 5% CO_2_ for 24 h. The cells were then washed twice with PBS, trypsinized, collected, and centrifuged at 300 × g for 5 min at room temperature. The cell pellet was resuspended, and apoptosis was assessed using the Annexin V-FITC/Propidium Iodide Apoptosis Detection Kit (Invitrogen), following the manufacturer’s instructions. Samples were analyzed by flow cytometry. For the spheroid apoptosis assay, SKOV-3 spheroids (5 days of formation at 37 °C with 5% CO_2_) were treated with the formulations and incubated for 3 days under the same conditions. Spheroids were then dissociated using trypsin with gentle repetitive pipetting. After dissociation, the cells were centrifuged and resuspended in Annexin V binding buffer. The Annexin V-FITC/Propidium Iodide apoptosis assay was performed following the manufacturer’s instructions, and cells were analyzed by flow cytometry (Fig. S9). For activated caspase staining, SKOV-3 cells were seeded on Lab-Tek 4 well chambered cover glass at a density of 5 × 10^4^ cells/mL. After 24 h, cells were transfected with SNP-DOX and SNP-DOX with MDR1-siRNA (0.5 mg/mL JBNt) and incubated at 37 °C and 5% CO2 for 24 h. Incucyte Caspase-3/7 Dyes for Apoptosis (Fisher Scientific) were added to each well following the manufacturer’s recommendations. Live cells were washed carefully with PBS before imaging. A Nikon A1 confocal laser-scanning microscope was used for fluorescence imaging. SKOV-3 spheroids (5 days, 37 °C and 5% CO_2_) were transfected with SNP-DOX and SNP-DOX with MDR1-siRNA (0.5 mg/mL JBNt) and then incubated at 37 °C and 5% CO2 for 4 days. Then the Incucyte Caspase-3/7 Dyes for Apoptosis were added to each well following the manufacturer’s recommendations.

#### 4.2.8. Reverse Transcriptase qPCR

SKOV-3 cells were seeded in 24-well plates and transfected with SNP-DOX, SNP-DOX-neg siRNA, SNP-DOX-MDR1 siRNA, or a no-treatment negative control, all diluted in McCoy’s 5A medium. The SNP-DOX group consisted of JBNt-DOX sonicated without siRNA. SNP-DOX-MDR1 siRNA was prepared using ON-TARGETplus Human ABCB1 siRNA SMARTpool (Horizon Discovery). SNP-DOX-neg siRNA was prepared using AllStars Negative Control siRNA (Qiagen). The no-treatment negative control consisted of medium alone. Cells were transfected for 24 h, achieving a final DOX concentration of 8 μg/mL and a final siRNA concentration of 50 nM. Then, total RNA was extracted using the Aurum Total RNA Mini Kit (Bio-Rad) according to the manufacturer’s instructions. For qPCR, primers were designed using GenBank and ordered from IDT for MDR1, caspase-3, and BCL-2 (Supplemtary Table S1). 18srRNA was used as a housekeeping gene. qPCR was carried out using SYBR green master mix (Bio-Rad) using the manufacturer’s recommended master mix conditions and thermocycler protocols with an annealing temperature of 60° C with a CFX Connect Real-Time thermal cycler (Bio-Rad). Relative mRNA expression was determined using the Delta Delta Ct method in addition to analysis using the CFX Maestro software (Bio-Rad).

#### 4.2.9. Endosomal escape

Human ovarian cancer SKOV-3 cells were seeded on glass-bottom confocal dishes at a density of 1 × 10^5^ cells per well and cultured overnight in McCoy’s 5A medium containing 10% fetal bovine serum (FBS) and 1% penicillin–streptomycin at 37 °C in a humidified 5% CO_2_ incubator. The following day, cells were treated with SNP–DOX–AF488 siRNA or LNP-DOX–AF488 siRNA formulations (Lipofectamine 2000 complexed with DOX and AF488-siRNA), while untreated cells were used as a control. After treatment, cells were gently washed with PBS and stained with LysoTracker^™^ Red DND-99 to visualize late endosomes and lysosomes. Nuclei were then counterstained with DAPI. After staining, cells were fixed with 4% paraformaldehyde. Images were acquired using a confocal laser scanning microscope with sequential scanning to prevent spectral overlap. Co-localization of AF488-labeled siRNA (green) with LysoTracker Red was analyzed using the Coloc2 plugin in ImageJ. Manders’ coefficients were calculated to quantify nanoparticle–lysosome co-localization, and Pearson’s correlation coefficients were used to assess signal overlap, with lower R values indicating enhanced cytosolic release of siRNA.

#### 4.2.10. Animal study

All animal procedures were conducted in accordance with protocols approved by the Institutional Animal Care and Use Committee (IACUC) of the University of Connecticut (Protocol No. A25–030). Female NU/J mice (10–12 weeks old; strain 002019) were purchased from The Jackson Laboratory (Bar Harbor, ME, USA) and used for all in vivo experiments, including treatment and biodistribution studies. Mice were randomly assigned to experimental groups. Animals were housed in a specific pathogen-free facility under controlled environmental conditions (25 ± 2 °C, 55% relative humidity) with a 12-h light/dark cycle and provided food and water ad libitum, in compliance with institutional IACUC guidelines.

#### 4.2.11. In vivo biodistribution study

Female NU/J mice (10–12 weeks old) were subcutaneously inoculated with 5 × 10^6^ SKOV-3 cells suspended in a 1:1 (v/v) mixture of cell suspension and Matrigel (Corning, #354234). Tumors were allowed to establish and grow for approximately 4 weeks until reaching a volume of ~200 mm^3^. For biodistribution analysis, mice were intravenously injected with saline or SNP–DOX–siRNA (DOX 0.86 mg/kg, siRNA 1.2 mg/kg per injection). The 0.86 mg/kg DOX dose was selected as a low-dose, dose-sparing regimen based on our previous JBNt-derived nanoparticle study using the same DOX dose for in vivo tumor delivery [11]. Three days after administration, major organs (heart, spleen, liver, kidney, and lung) and tumor tissues were harvested for ex vivo quantification of the relative fluorescence intensity of DOX and AF647-tagged siRNA (n = 3).

#### 4.2.12. In vivo antitumor efficacy of SNP-DOX-siRNA in SKOV-3 tumor models

Following subcutaneous inoculation with 5 × 10^6^ SKOV-3 cells, tumors were allowed to grow for approximately 4 weeks until reaching a volume of ~200 mm^3^, which was defined a priori as the baseline tumor burden for treatment initiation. Tumor volume was measured using digital calipers and calculated as V = ½ (length × width^2^). Mice were then randomly assigned to treatment groups (n = 6 per group), including saline and SNP-DOX-MDR1 siRNA, with group allocation balanced to ensure comparable mean tumor volumes at baseline. Investigators performing longitudinal tumor measurements were blinded to treatment allocation. Mice received intravenous injections (150 μL per dose) every six days for a total treatment duration of 21 days, corresponding to four administrations per mouse. The DOX dose was 0.86 mg/kg per injection, and the siRNA dose was 1.2 mg/kg per injection. Tumor growth curves were generated based on serial volume measurements throughout the study period.

#### 4.2.13. Cryosection preparation and staining

Excised tissues were rinsed with PBS, fixed in 4 % paraformaldehyde for 24 h at 4 °C, and cryoprotected in 30 % sucrose until equilibrated. Samples were embedded in OCT compound, frozen, and sectioned at 8–10 μm thickness using a cryostat. For tumor sections, immunofluorescence staining was performed using anti-cleaved caspase-3 antibody (1:200, Cell Signaling Technology) followed by Alexa Fluor-conjugated secondary antibody. TUNEL assay (Thermo Fisher Scientific) was used to detect DNA fragmentation. Nuclei were counterstained with DAPI, and images were acquired on a CLSM. Cryosectioned organs were stained with hematoxylin and eosin (H&E) and examined under a light microscope to evaluate potential tissue toxicity.

#### 4.2.14. Whole blood analysis

Peripheral whole blood was collected from mice at the initial and terminal endpoint of the in vivo treatment study and transferred into MiniCollect K2E K2EDTA blood collection tubes for hematological analysis. Tubes were gently inverted after collection to ensure proper anticoagulant mixing and to prevent clotting. Samples were maintained at 2–8 °C and submitted to IDEXX BioAnalytics for complete blood count (CBC) analysis. Hematological parameters, including white blood cell count, red blood cell count, hemoglobin, hematocrit, platelet count, and leukocyte differential counts, were analyzed. CBC results are presented in Supplementary Table. S2.

#### 4.2.15. Statistical analysis

Statistical analyses were performed using GraphPad Prism. Data are presented as mean ± SEM unless otherwise stated. For comparisons between two groups, an unpaired two-tailed Student’s t-test was used. For comparisons among three or more groups, one-way analysis of variance (ANOVA) was performed, followed by Tukey’s multiple comparisons test. Longitudinal tumor growth data (Fig. 7D) were analyzed by two-way ANOVA. A P value < 0.05 was considered statistically significant. Sample sizes for each experiment are indicated in the corresponding figure legends.

## Supplementary Material

1

## Data Availability

Data will be made available on request.

## Appendix A. Supplementary data

Supplementary data to this article can be found online at https://doi.org/10.1016/j.mtadv.2026.100845.
