# Supplementary material for: Enhanced endosomal escape for nanoparticle-enabled co-delivery of doxorubicin and siRNA to overcome multidrug resistance
Source: Mater Today Adv. Author manuscript; Available in PMC 2026 Jul 2. (PMC13322303; doi:10.1016/j.mtadv.2026.100845)
Supplement: 1 [file NIHMS2188800-supplement-1.docx]

**Enhanced Endosomal Escape for Nanoparticle-Enabled Co-Delivery of Doxorubicin and siRNA to Overcome Multidrug Resistance**

^†^Jin Zhai^1^, ^†^Allison Surian^1^, Trystin Cote^1^, Wuxia Zhang^1^, Bao-Toan Dang^1^, Yuan Wang^1^, Qianyu Chen^1^, Kazunori Hoshino^1^, *Jinhyung Lee^1^, *Yupeng Chen^1^

^1^Department of Biomedical Engineering, University of Connecticut, Storrs, CT 06269, USA

^†^These authors contributed equally to this work.

***Authors to whom correspondence should be addressed.**

Dr. Jinhyung Lee: jinhyung.lee@uconn.edu

Dr. Yupeng Chen: yupeng.chen@uconn.edu

**Keywords**

Nanoparticle; Multidrug resistance; Co-delivery; Anticancer therapy; Endosomal escape

**Supplementary Information Guide**

Supplementary Figure S1. Characterization of SNP-DOX-siRNA candidates.

Supplementary Figure S2. Aspect ratio of SNP-DOX-siRNA.

Supplementary Figure S3. Stability of SNP-DOX-siRNA.

Supplementary Figure S4. Encapsulation efficiency of siRNA in SNP-DOX-siRNA.

Supplementary Figure S5. Time-dependent growth of SKOV-3 cancer spheroids.

Supplementary Figure S6. Flow cytometric gating strategy for SNP-DOX-siRNA delivery.

Supplementary Figure S7. Western blot analysis of P-gp protein expression.

Supplementary Figure S8. Flow cytometry analysis of P-gp protein expression.

Supplementary Figure S9. Flow cytometric gating of apoptosis assay.

Supplementary Figure S10. Ex vivo biodistribution of SNP-DOX-siRNAs in mice bearing SKOV-3 tumors.

Supplementary Figure S11. Complete blood count (CBC) analysis of SNP-DOX-siRNA.

Supplementary Table S1. Primer sequence of quantitative polymerase chain reaction.

Supplementary Table S2. Complete blood count (CBC) study.

**Supplementary Fig 1.**


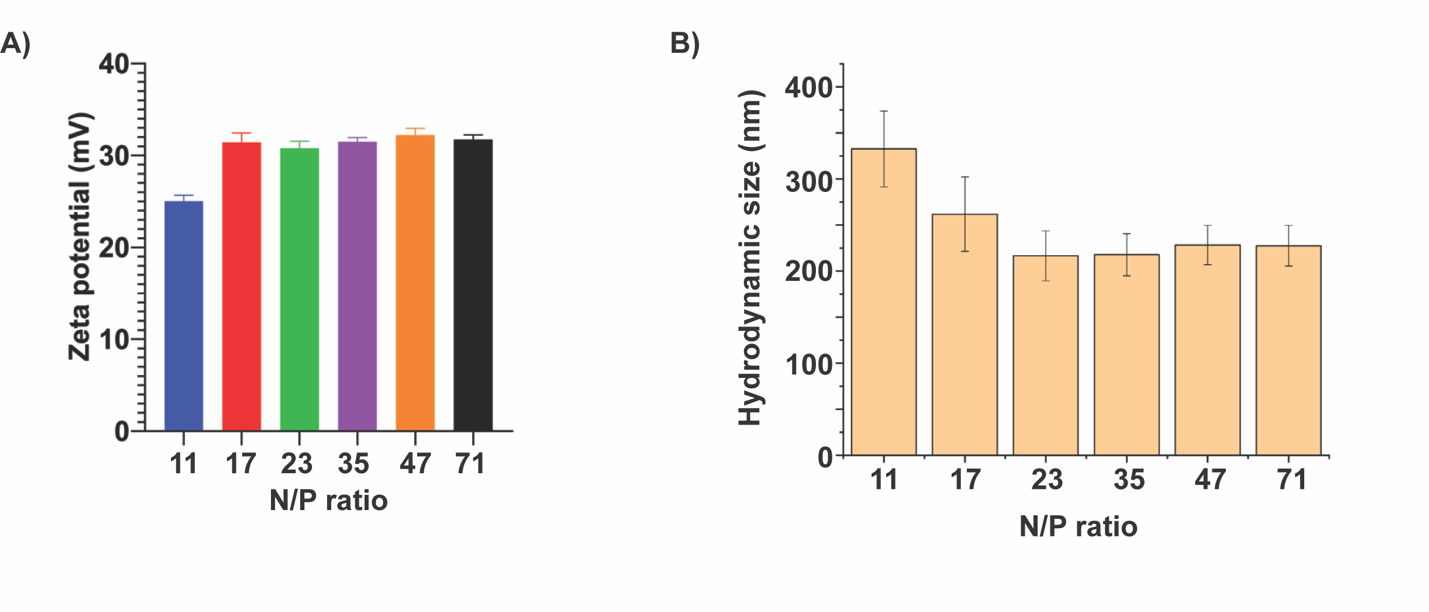


**Figure S1. Characterization of SNP-DOX-siRNA candidates.** (A) Zeta potential of SNP-DOX-siRNA formulations at varying N/P ratios, defined as the molar ratio of positively charged amine (N) groups in JBNt to negatively charged phosphate (P) groups in siRNA. (B) Hydrodynamic diameter of SNP-DOX-siRNA formulations at varying N/P ratios. N=3, mean ± SEM.

**Supplementary Fig 2.**


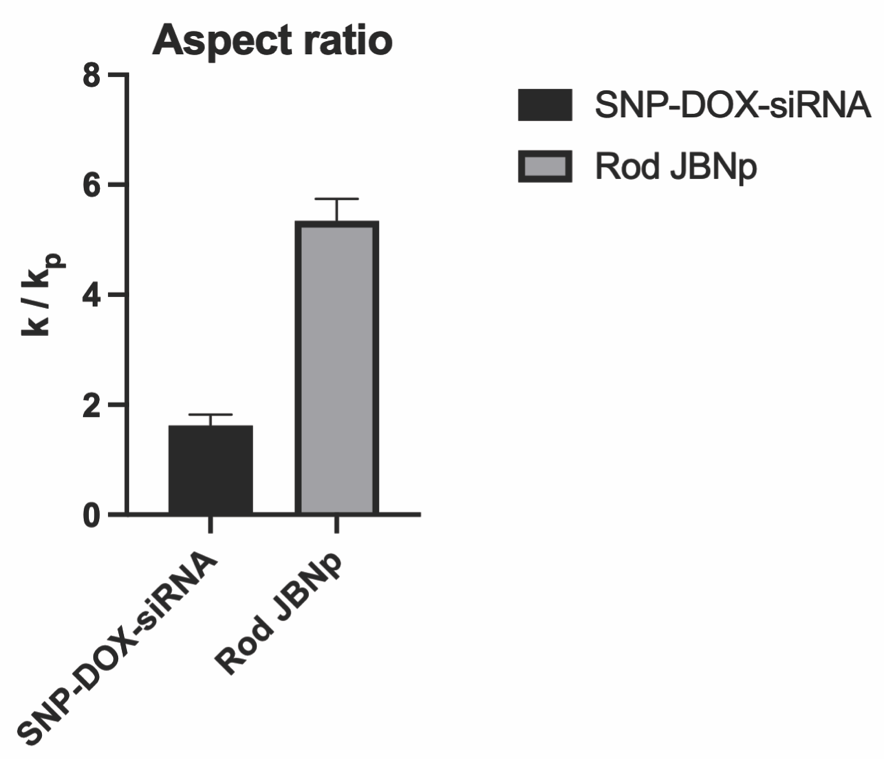


**Figure S2.** **Aspect ratio of SNP-DOX-siRNA compared with rod-shaped JBNp.** The aspect ratio (AR) was calculated from TEM images as the length (k) divided by the width (kₚ), with measurements obtained from n = 12 particles. Previously, rod-shaped JBNp exhibited an AR of 5.3, with a length of 126.3 ± 13.9 nm and a width of 26.7 ± 2.5 nm [11]. In contrast, SNP-DOX-siRNA displayed an AR of approximately 1.6. Here, k/kₚ represents length divided by width.

**Supplementary Fig 3.**


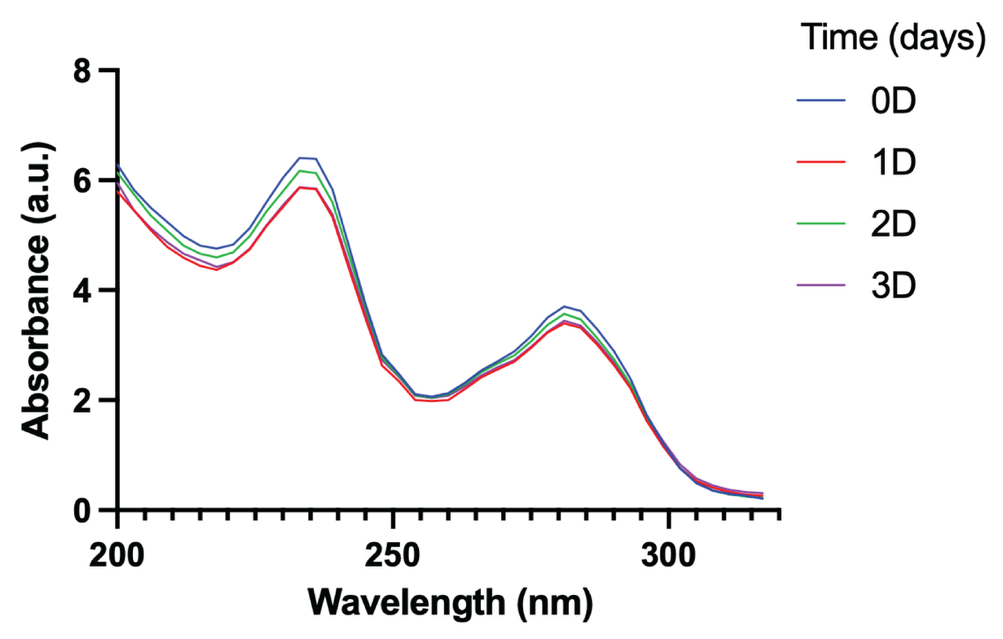


**Figure S3. Stability of SNP-DOX-siRNA.** UV-Vis absorbance spectra of SNP-DOX-siRNA over a 3-day period demonstrated time-dependent stability and preservation of the characteristic absorbance profile of the nanoparticle formulation.

**Supplementary Fig 4.**


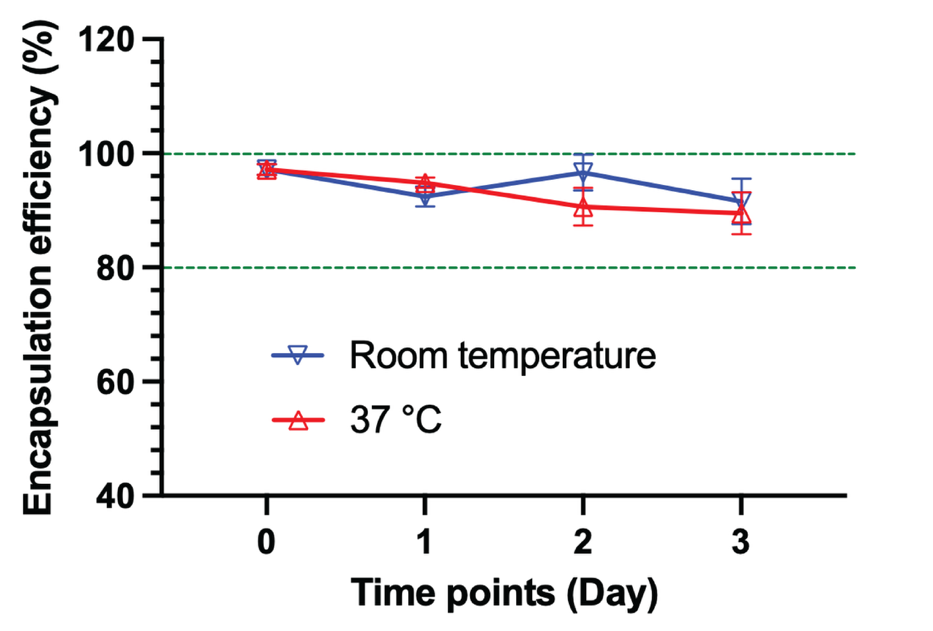


**Figure S4. Encapsulation efficiency and retention of siRNA in SNP-DOX-siRNA nanoparticles.** RiboGreen analysis of SNP-DOX-siRNA over a 3-day period at room temperature and 37 °C demonstrated sustained siRNA retention and stability within the SNP-DOX-siRNA complex (N = 3, mean ± SEM).

**Supplementary Fig 5.**


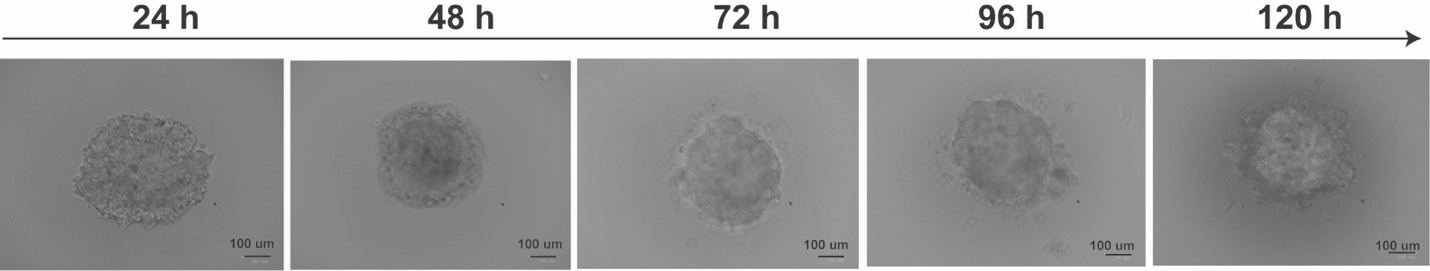


**Figure S5.** **Time-dependent growth of SKOV-3 cancer spheroids.** SKOV-3 cells were seeded in 96-well Nunclon Sphera round-bottom plates at a density of 2 × 10³ cells per well. Cells were cultured for 5 days to allow formation of compact and uniform spheroids prior to subsequent experiments.

**Supplementary Fig 6.**

**
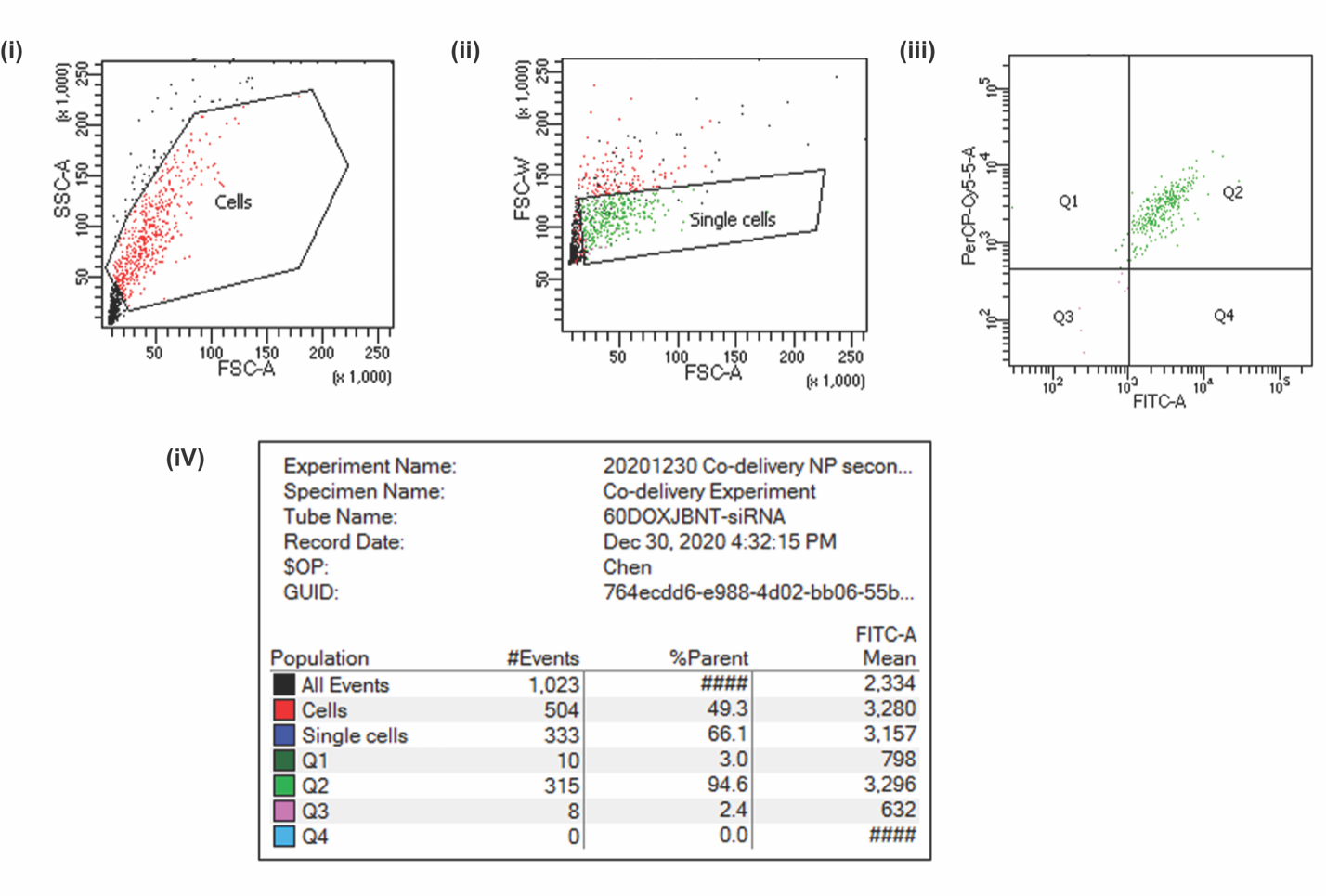
**

**Figure S6.** **Flow cytometric gating strategy for SNP-DOX-siRNA delivery.** Representative gating strategy used to characterize SKOV-3 cells following transfection with SNP-DOX–AF488–tagged siRNA. (i) Identification of the cell population based on forward and side scatter. (ii) Doublet exclusion to gate single cells. (iii) Fluorescence gating for FITC-A and PerCP-Cy5-5A signals. (iv) Quantification of events FITC-A signal and PE-Cy5-5A signal.

**Supplementary Fig 7.**

**
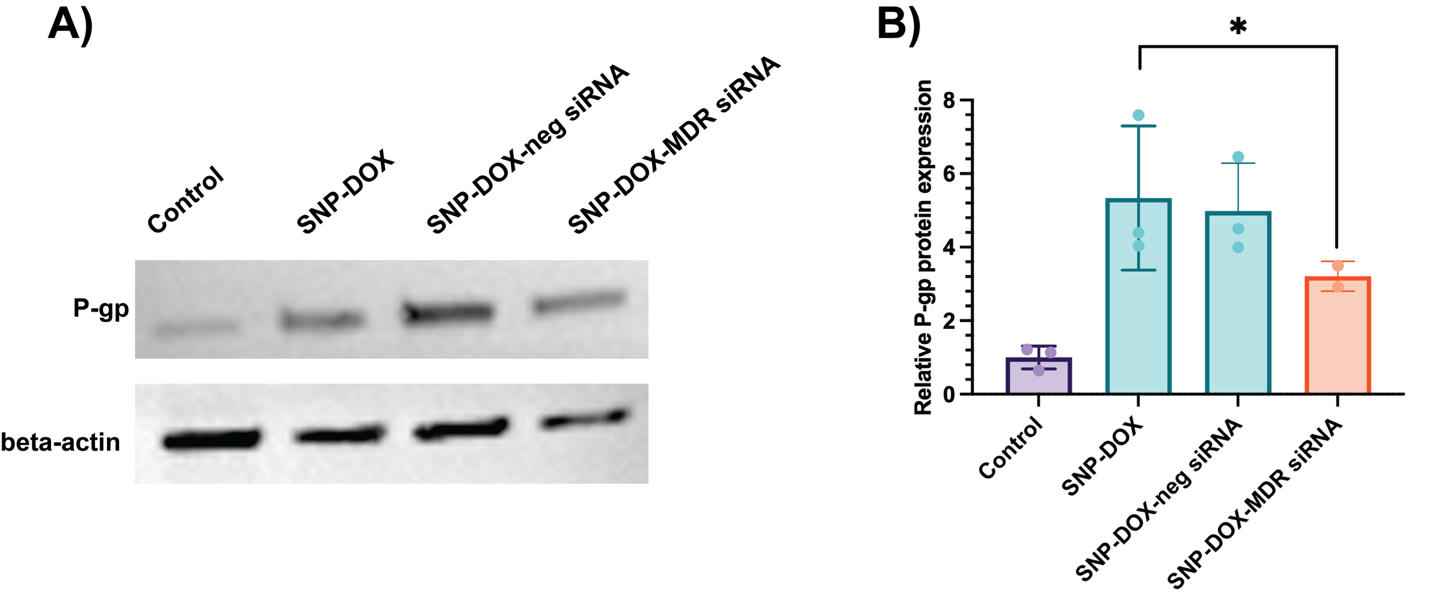
**

**Figure S7. Relative P-glycoprotein (P-gp) protein expression levels.** Representative Western blot images are shown in panel (a), including P-gp expression in the control, SNP-DOX, SNP-DOX-neg siRNA, and SNP-DOX-MDR siRNA groups, with β-actin used as the loading control. Quantitative analysis of the western blot data is shown in panel (b), demonstrating that SNP-DOX-MDR siRNA significantly reduced P-gp protein expression compared with the SNP-DOX control group (N = 3, mean ± SEM, *P < 0.05).

**Supplementary Fig 8.**


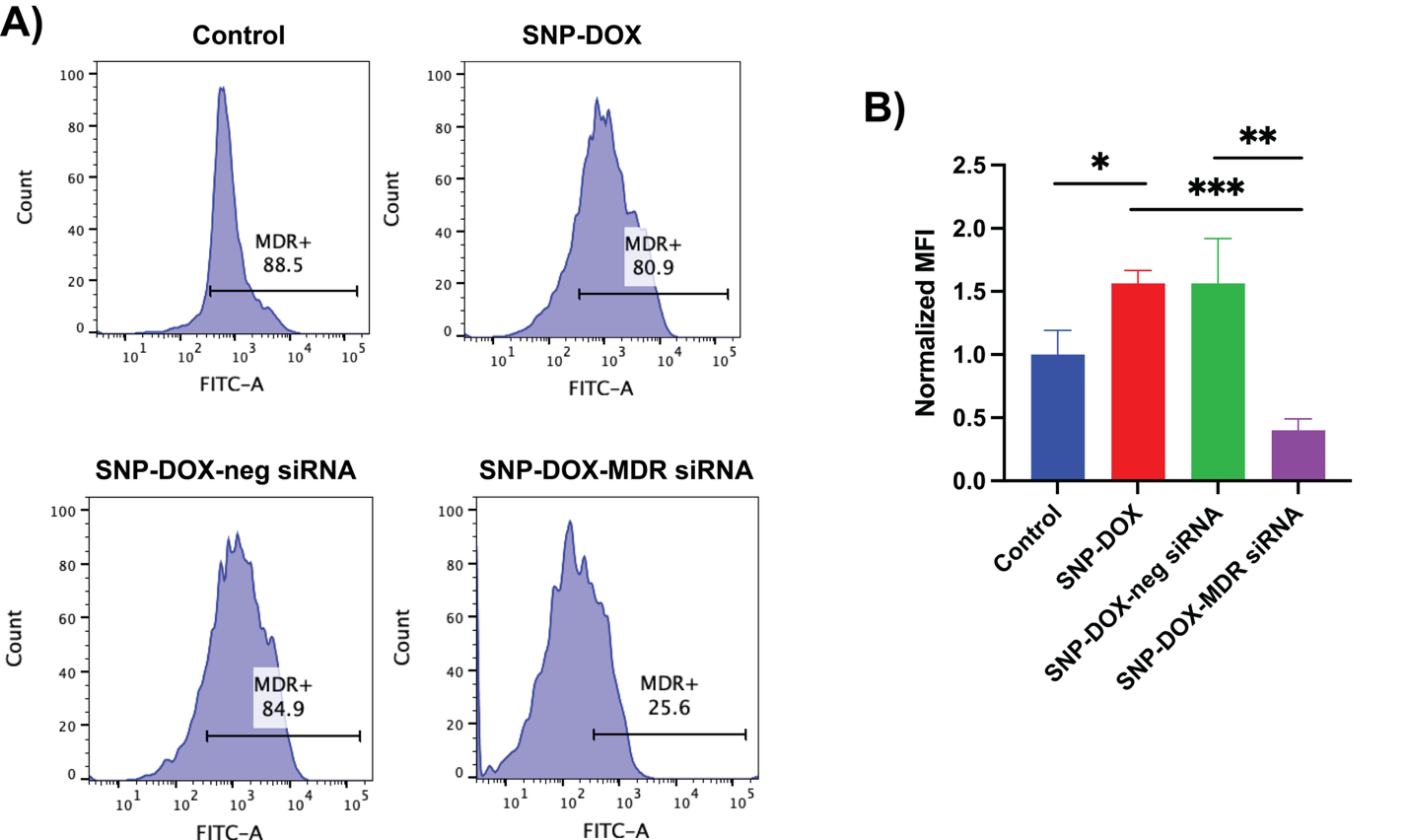


**Fig. S8.** **Flow cytometry analysis of P-gp protein expression.** (A) Quantification of the percentage of MDR/P-gp-positive cells by flow cytometry demonstrated that SNP-DOX-MDR siRNA significantly decreased MDR/P-gp protein expression compared with the SNP-DOX and SNP-DOX-neg siRNA groups, as measured using an anti-MDR/P-gp primary antibody and an AF488-conjugated secondary antibody (N = 3). (B) Mean fluorescence intensity (MFI) analysis by flow cytometry demonstrated that SNP-DOX-MDR siRNA significantly decreased MDR/P-gp protein expression compared with the SNP-DOX and SNP-DOX-neg siRNA groups, as measured using an anti-MDR/P-gp primary antibody and an AF488-conjugated secondary antibody (N = 3, mean ± SEM, *P < 0.05).*

**Supplementary Fig 9.**

**
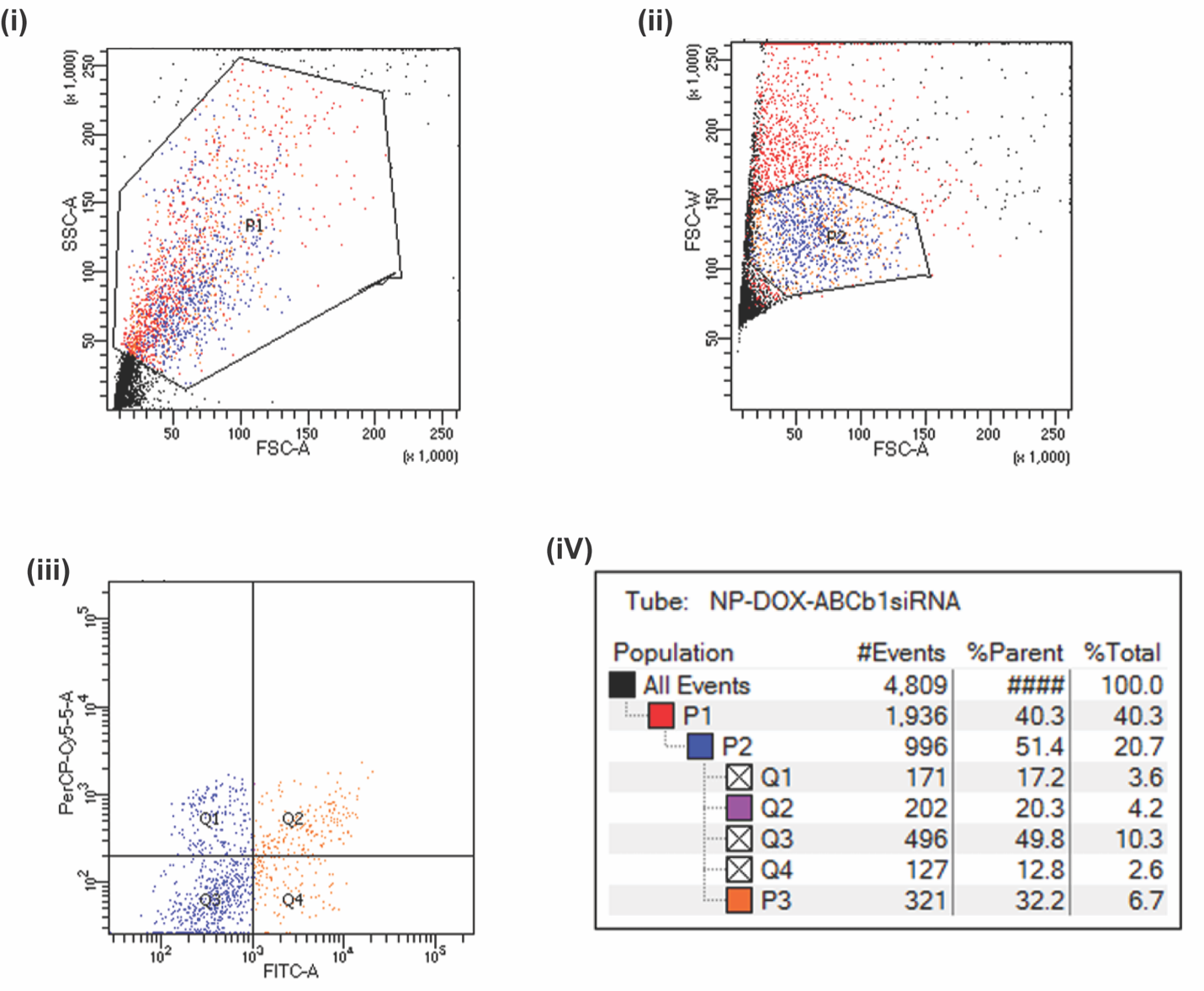
**

**Figure S9. Flow cytometric gating strategy for apoptosis analysis.** Representative gating strategy used to assess apoptosis in SKOV-3 cells following transfection with SNP-DOX–MDR (ABCB1) siRNA. (i) Identification of the cell population based on forward and side scatter. (ii) Doublet exclusion to gate single cells. (iii) Fluorescence gating for FITC-A and PerCP-Cy5-5A signals. (iv) Quantification of FITC-A– and PE-Cy5-5A–positive events.

**Supplementary Fig 10.**


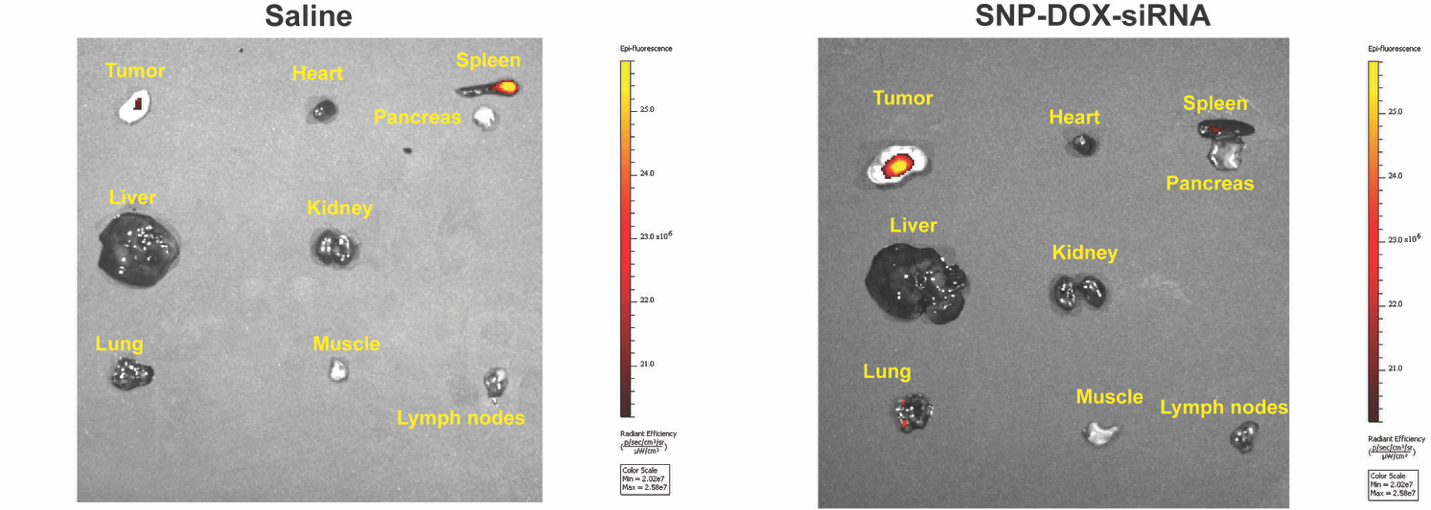


**Figure S10.** Ex vivo biodistribution of SNP-DOX- MDR1 siRNA in mice bearing SKOV-3 tumors. (A) Organs and tumor were harvested after 72 h after i.v. injection. In vivo imaging system (IVIS) was used to image tumor, heart, spleen, liver, kidney, pancreas, lung, muscle and lymph nodes. The color scale to the right indicates radiant efficiency.

**Supplementary Fig 11.**

**
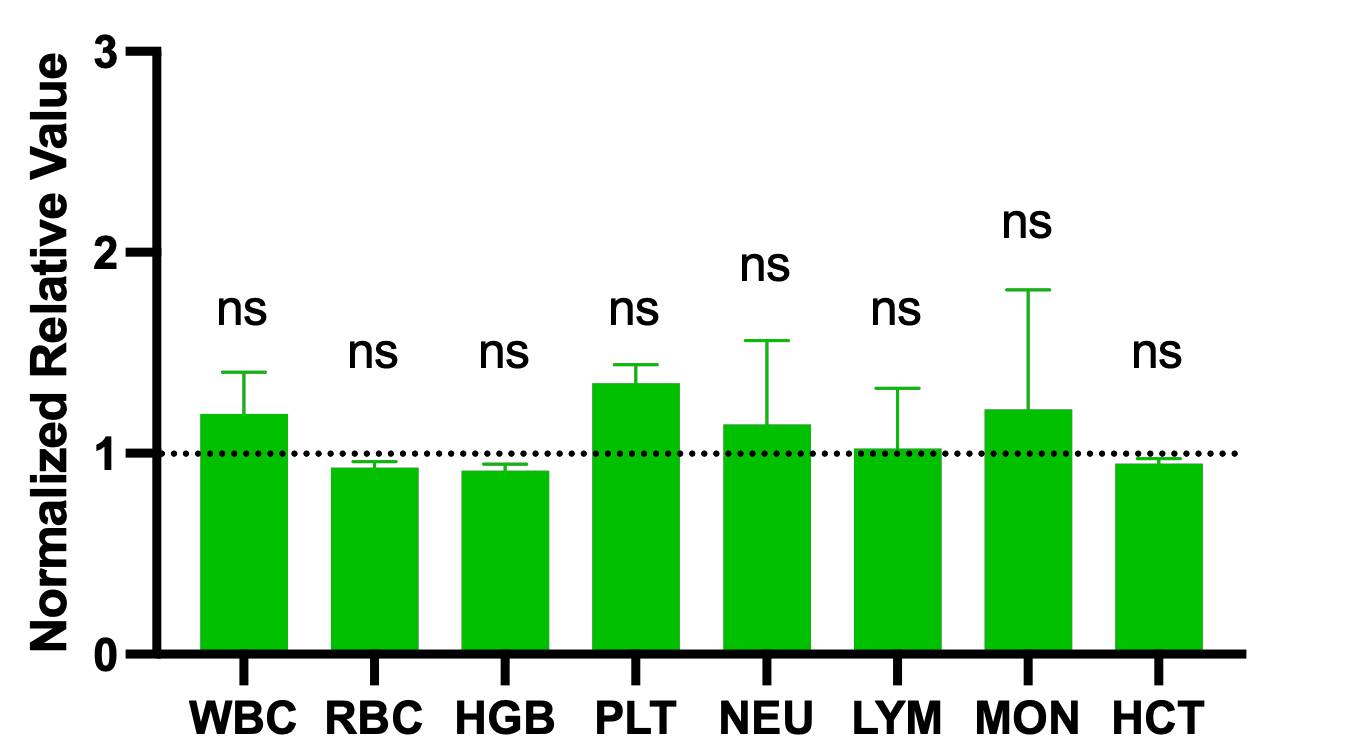
**

**Figure S11. Complete blood count (CBC) analysis of SNP-DOX-siRNA.** CBC analysis before and after systemic treatment with SNP-DOX-siRNA in SKOV-3 tumor-bearing mice. Hematological parameters were normalized to pre-treatment values and included white blood cells (WBC), red blood cells (RBC), hemoglobin (HGB), platelets (PLT), neutrophils (NEU), lymphocytes (LYM), monocytes (MON), and hematocrit (HCT). Data are presented as mean ± SEM (n = 5 per group, *P < 0.05, ns, not signficiant).

**Supplementary Tables**

**qPCR primer sequences**

| **Gene** | **Forward Primer** | **Reverse Primer** |
| --- | --- | --- |
| 18S rRNA | GTA ACC CGT TGA ACC CCA TT | CCA TCC AAT CGG TAG TAG CG |
| MDR1 | GGT CTG CCC CCA TCA AAT GA | ATA CTT CCT GCT AGG CCC CA |
| Caspase-3 | TTC AGA GGG GAT CGT TGT AGA AGT C | CAA GCT TGT CGG CAT ACT GTT TCA G |
| BCL-2 | AAA AAG GTT GGG GAG GTG CA | CCC GTT GCC CTA AAT GTC CT |

**Table S1. Primer sequences designed for a 60 °C annealing temperature**. Forward and reverse primer sequences used for qPCR.

**Complete blood count (CBC) analysis**

| Parameter | Baseline | After treatment |
| --- | --- | --- |
| White Blood cell (10^9^ cells / liter) | 3.92 $\pm$ 0.64 | 4.40 $\pm$ 0.48 |
| Red Blood Cell (10^12^ cells / liter) | 8.41 $\pm$ 0.28 | 8.42 $\pm$ 0.37 |
| Hemoglobin (g/l) | 11.83 $\pm$ 0.45 | 11.66 $\pm$ 0.38 |
| Platelet (10^9^ cells / liter) | 683.33 $\pm$ 82.26 | 966.17 $\pm$ 116.41 |
| Neutrophil (10^9^ cells / liter) | 1.12 $\pm$ 0.11 | 1.62 $\pm$ 0.36 |
| Lymphocyte (10^9^ cells / liter) | 2.62 $\pm$ 0.58 | 2.41 $\pm$ 0.41 |
| Monocyte (10^9^ cells / liter) | 0.17 $\pm$ 0.02 | 0.23 $\pm$ 0.12 |
| Hematocrit (%) | 38.01 $\pm$ 1.04 | 38.63 $\pm$ 1.39 |

**SNP-DOX-siRNA**

**Table S2. Result of the complete blood count (CBC) study.** (A) CBC values of SNP-DOX- siRNA including White blood cells (WBC), Red blood cells (RBC), Hemoglobin (HGB), Platelets (PLT), Neutrophil (NEU), Lymphocyte (LYM), Monocyte (MON), and Hematocrit (HCT), expressed as mean and standard error of the mean (SEM), evaluated in a group of five SKOV-3 tumor-bearing NU/J mice.
